# A Depletion of Stop Codons in lincRNA is Owing to Transfer of Selective Constraint from Coding Sequences

**DOI:** 10.1093/molbev/msz299

**Published:** 2019-12-16

**Authors:** Liam Abrahams, Laurence D Hurst

**Affiliations:** Milner Centre for Evolution, Department of Biology and Biochemistry, University of Bath, Bath, United Kingdom

**Keywords:** transfer selection, lincRNA, exonic splice enhancer, ESE, stop codon, sequence evolution

## Abstract

Although the constraints on a gene’s sequence are often assumed to reflect the functioning of that gene, here we propose *transfer selection*, a constraint operating on one class of genes transferred to another, mediated by shared binding factors. We show that such transfer can explain an otherwise paradoxical depletion of stop codons in long intergenic noncoding RNAs (lincRNAs). Serine/arginine-rich proteins direct the splicing machinery by binding exonic splice enhancers (ESEs) in immature mRNA. As coding exons cannot contain stop codons in one reading frame, stop codons should be rare within ESEs. We confirm that the stop codon density (SCD) in ESE motifs is low, even accounting for nucleotide biases. Given that serine/arginine-rich proteins binding ESEs also facilitate lincRNA splicing, a low SCD could transfer to lincRNAs. As predicted, multiexon lincRNA exons are depleted in stop codons, a result not explained by open reading frame (ORF) contamination. Consistent with transfer selection, stop codon depletion in lincRNAs is most acute in exonic regions with the highest ESE density, disappears when ESEs are masked, is consistent with stop codon usage skews in ESEs, and is diminished in both single-exon lincRNAs and introns. Owing to low SCD, the maximum lengths of pseudo-ORFs frequently exceed null expectations. This has implications for ORF annotation and the evolution of de novo protein-coding genes from lincRNAs. We conclude that not all constraints operating on genes need be explained by the functioning of the gene but may instead be transferred owing to shared binding factors.

## Introduction

When considering the evolution of a gene or protein we assume, often implicitly, that sequence constraints within that gene are important in terms of the functioning of its RNA/protein products. For example, when we observe constraint on a protein domain within any given protein, we trivially assume it to be a result of the domain being important for the function of that protein. The same logic extends beyond protein motifs to RNA level features such as microRNA pairing sites. The assumption that features of genes or proteins exist to enable the functioning of that gene or protein appears so self-evidently correct that it is difficult to comprehend that there may be selectively constrained features of genes that do not reflect the functioning of the gene in question, except for overlapping genes. In this article, we suggest that compositional patterns observed in some genes may instead be explained by a transfer of a selective constraint from one class of gene to another. We present an exemplar theoretical instance and show that it makes correct predictions of otherwise paradoxical sequence features.

Our exemplar considers the stop codon density (SCD) in long intergenic noncoding RNAs (lincRNAs). We define codon density as the number of nucleotide positions constituted by the codon in question in any frame of a given sequence, divided by the total number of nucleotides in the sequence. For example, in the sequence AGATAGGGGA, the GGA codon (AGATAGG**GGA**) has a density of 0.3. By counting each nucleotide within the queried sequence only once, the density is bound by the limits 0 and 1 (e.g., the density of the codon GGG in the same sequence AGATA**GGGG**A is 0.4). We can extend our density calculation to codon sets, by considering groupings of more than one codon whose density we calculate together as per single codon cases. For example, the dicodon set {GAT, GGG} defines 7/10 positions (A**GAT**A**GGGG**A) and has a density of 0.7. Thus, we define SCD as the positions composed of the tricodon set {TAA, TAG, TGA}. The sequence GG**TGATAA**CA, for example, has SCD equal to 0.6.

Unlike coding sequence (CDS) that is constrained to one in-frame stop codon per sequence, lincRNAs have no comparable constraint. The SCD in lincRNAs should therefore be predictable from underlying nucleotide content. However, we argue that a particular mode of selection, which might be termed *transfer selection*, would result in lower stop codon usage than expected. Our argument is simple. Exonic splice enhancers (ESEs), typically short hexameric motifs occurring toward exon ends (within ≈70 bp of the splice site) (Berget 1995; Fairbrother et al. 2002; Fairbrother, Holste, et al. 2004; Carlini and Genut 2006; Parmley et al. 2006, 2007; Woolfe et al. 2010; Caceres and Hurst 2013) act as binding sites in the immature mRNA for serine/arginine-rich (SR) proteins to help direct the splice machinery. As ESEs overlap CDS, they cannot introduce an in-frame stop codon. Consequently, it seems highly likely that ESEs functioning in CDS are under selection to contain no or few stop codons. If SR proteins bind the same or similar ESEs in multiexon coding and noncoding transcripts, the need to employ ESEs in lincRNAs should mean a depletion of stop codons in CDS ESEs transfers to lincRNA ESEs, despite stop codons in lincRNA having no translational function. In short, the binding preferences of SR proteins in CDS may transfer a necessary constraint operating on CDS to an unnecessary and otherwise paradoxical sequence constraint operating in noncoding sequences.

Many of the assumptions of our model are robust. First, lincRNA transcripts containing introns are processed similarly to protein-coding pre-mRNA transcripts (reviewed by Will and Luhrmann [2011] and De Conti et al. [2013]). Although SR protein binding is reported to be ≈30% less efficient in lincRNA than in protein-coding exons, evidence suggests that the same SR proteins bind both gene classes as the binding of SR proteins SRSF2, SRSF5, and SRSF6 in lincRNA all improve splicing efficiency (Krchnakova et al. 2019). Second, ESEs are under purifying selection in both CDS and lincRNA, indicative of functionality. In CDS, this is illustrated by decreased rates of evolution at both synonymous (Fairbrother et al. 2002; Carlini and Genut 2006; Chamary et al. 2006; Parmley et al. 2006; Parmley and Hurst 2007; Sterne-Weiler et al. 2011; Caceres and Hurst 2013; Savisaar and Hurst 2018) and nonsynonymous sites (Parmley et al. 2006, 2007) and the relative lack of single-nucleotide polymorphisms (Majewski and Ott 2002; Fairbrother, Holste, et al. 2004; Carlini and Genut 2006; Caceres and Hurst 2013) within ESEs. This selection is not modest and, indeed, the proportion of exonic sequence devoted to governing splicing, predominantly moderated by selection for ESEs, predicts the rate of human protein evolution as well as the amount a gene is expressed, the phylogenetically universal best predictor (Parmley et al. 2007). Similarly, purifying selection on ESEs is thought to explain most lincRNA constraint (Schuler et al. 2014; Haerty and Ponting 2015).

To test this model of transfer selection, we start by asking whether the SCD in ESE motifs is unusually low. We find this to be the case, even when controlling for the nucleotide composition of ESEs. We then ask whether, in contrast to a priori expectation, lincRNA sequences are also relatively depleted in stop codons and, if so, whether ESEs are the cause. We show that lincRNAs do contain fewer stop codons than expected given their nucleotide content. We provide several lines of evidence to support the hypothesis that this is due to the presence of ESEs and not open reading frame (ORF) sequence contamination.

Selective avoidance of stop codons could, at first sight, be misinterpreted as evidence that any given lincRNA is an unrecognized coding gene. As the low density of stop codons in lincRNAs ensures that the longest possible ORF is longer than expected under null models, our finding has ramifications for transcript annotation. We show that the typically used threshold of minimal ORF size (300 bp) causes a high (≈10%) false-positive rate if used in isolation. Although the dearth of stop codons could confuse annotation, it might also have consequences for de novo gene origination via erroneous translation of noncoding RNA as accidental peptides can be longer than expected.

## Results

If our model of transfer selection has validity, results must be consistent with several predictions. First, for any motif that functions within CDS, the protein-coding constraint requires it to contain no stop codons in one of the three reading frames. Thus, stop codons should be relatively rare in ESE motifs that have to reside in CDS. The same need not be true of motifs that function exclusively in introns or noncoding exons. Second, any rarity should be specific to the set of stop codons and not peculiarities resulting from motif set choice or motif functionality. Third, stop codons should also be depleted in lincRNA sequences after accounting for their nucleotide content, this depletion being attributable to ESEs. We test each of these predictions.

### ESEs Are Depleted in Stop Codons

To address the first prediction, we first consider the SCD in the “gold-standard” (low false-positive) INT3 ESE motif set (*N* = 84 hexamers), for which each motif was identified in at least three of four high-throughput ESE data sets (Caceres and Hurst 2013) (see Materials and Methods for an overview of how each ESE set was derived). The raw INT3 SCD is 0.054, lower than the SCD of 0.094 for the 4,012 possible hexamers not found in the INT3 set. This low SCD in the INT3 set is significantly lower than SCDs of 10,000 iterations of 84 hexamers randomly sampled from the pool of all possible 4,096 hexamers (*P* ≈ 0.034, one-tailed empirical *P* value). Thus, to a first approximation, stop codons appear depleted in the true ESE motifs.

### ESEs Are Significantly Depleted in Stop Codons after Controlling for Nucleotide Content

The above result is prima facie evidence that ESE motifs are unusual in having a low SCD. However, it could also be owing to underlying nucleotide biases within the set of ESEs. If so, ESEs should also be depleted of codons of similar nucleotide content to the stop codons. To address whether the low SCD of ESEs reflects an avoidance specific to the stop codons, we have to control for both the nucleotide content of the stop codons and nucleotide content of the ESE motifs.

**FIg. 1.**
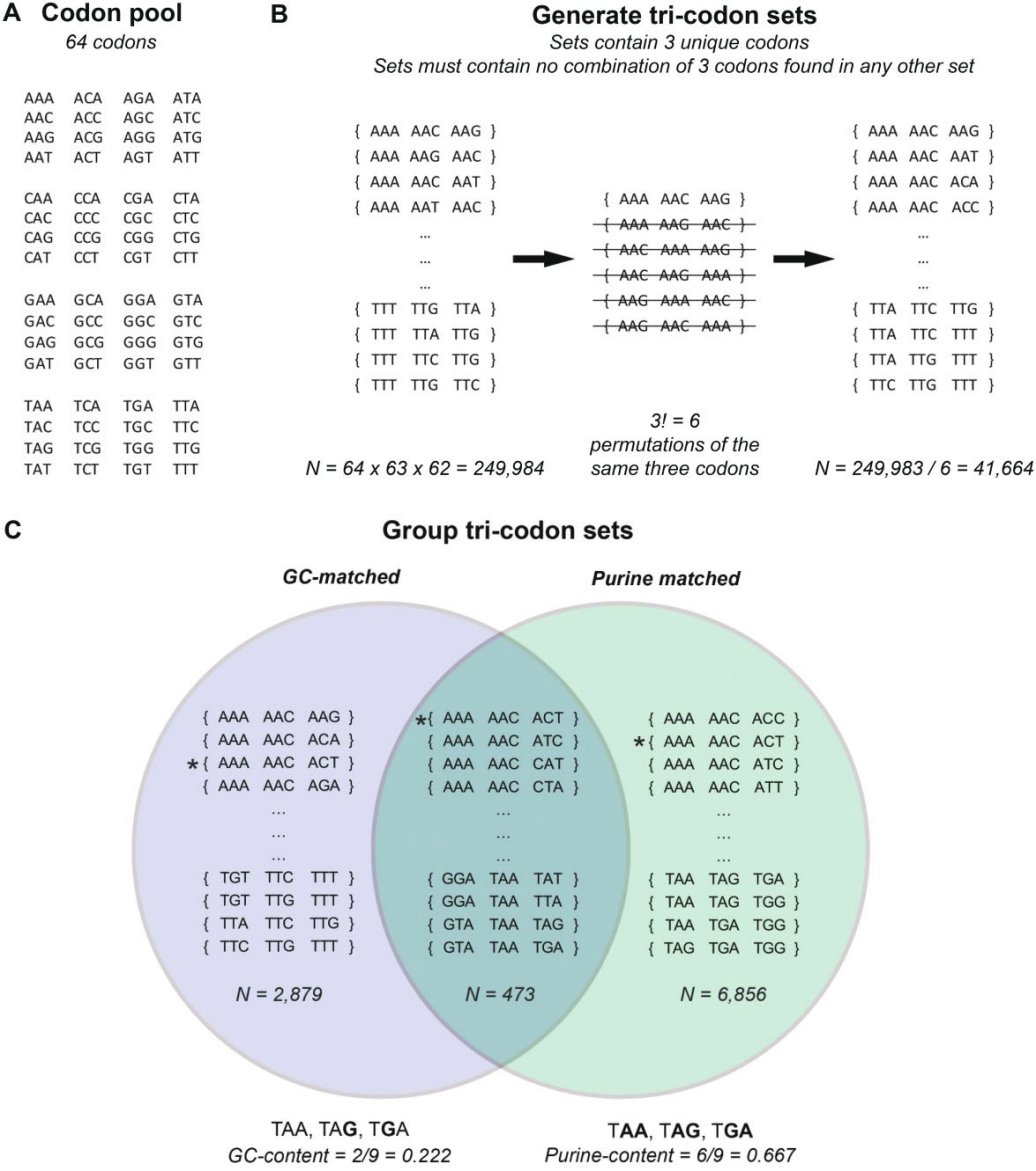
Overview of how the tricodon sets were derived. (*A*) Every codon was considered (*N* = 64). (*B*) Every possible permutation of three codons was generated, ensuring each permutation contained three unique codons, leaving *N* = 64 × 63 × 62 = 249,984 sets. For each grouping of three unique codons, there exists 3! = 6 possible permutations of the three codons. Codon sets with the same three codons, just in a different order, were considered to be the same codon set, and so duplicates were removed leaving *N* = 249,984/6 = 41,664 codon sets. (*C*) The codon sets from (*B*) were then grouped. The first set contains codon sets with identical net GC content to the stop codons (GC = 0.222, *N* = 2,879). A second contained codon sets with identical net purine content as the stop codons (purine = 0.667, *N* = 6,856). Finally, a set comprising the intersection of both GC- and purine-matched sets was generated (*N* = 473). The example codon set {AAA, AAC, ACT} has both equal GC and purine content to the stop codons and is highlighted by the *.

To control for the nucleotide content of the stop codons, we compiled codon sets that are compositionally matched to the stop codon set (see fig. 1*A*–*C*, and Materials and Methods). We start by considering the 2,879 GC-matched tricodon sets (i.e., with GC content = 0.222, the same as the stop codon set). To test whether the stop codons specifically are underemployed in ESEs, we also have to control for ESE nucleotide content. We therefore generated 10,000 dinucleotide-matched pseudo-ESE motif sets (*N* = 84 pseudo-motifs per iteration matching the number of INT3 motifs). For any given codon set, we can then calculate a fold-enrichment (FE) score (see Materials and Methods) that gives the relative enrichment of a given codon set in the true ESEs while accounting for underlying ESE nucleotide content. FE > 0 implies enrichment, FE < 0 implies depletion, and FE ≈ 0 reflects null.

**FIg. 2.**
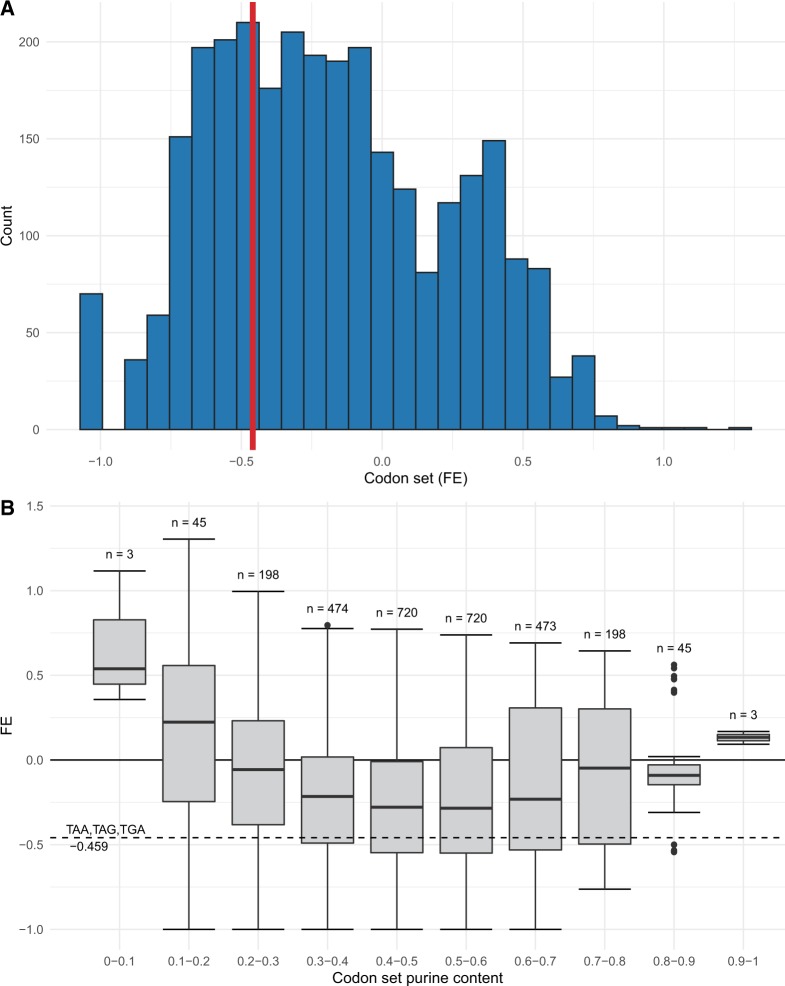
Comparisons of FE scores of the stop codon set. (*A*) Histogram showing the FE scores of codon sets containing three unique codons with identical GC content to the stop codon set (GC = 0.222) in INT3 ESEs. The stop codon set highlighted by the vertical line. When controlling for the dinucleotide-content of ESEs, the FE of the stop codon set is highly depleted compared with GC-matched codon sets and falls toward the lower tail of the distribution of FE scores. (*B*) Boxplots of FE scores for tricodon sets with GC content equal to that of the set of stop codons, grouped by purine content. Not only is the FE of the stop codon set (dotted horizontal line) reduced when compared with GC-matched codon sets, it is significantly reduced (*P* = 5.484 × 10^−14^, one-tailed exact binomial test) when compared with sets also containing identical purine content (purine content grouping 0.6–0.7, *N* = 473).

If stop codons are depleted in the true set of ESEs, because they are stop codons, their FE should be lower than the FE of the GC-matched control codon sets. Conversely, if stop codons are depleted in ESEs because of the nucleotide content of stop codons and ESEs, their FE should be no lower than the FE of the GC-matched control codon sets. We find 2,018/2,879 = 70.09% of the GC-matched codon sets have a higher FE than for the stop codon set (*P* < 2.2 × 10^−16^, one-tailed exact binomial test, null probability of success = 0.5, fig. 2*A*), consistent with a depletion due to being stop codons.

A particular curiosity of the ESE motifs (and of INT3 ESEs more specifically) is that they are purine rich (mean number of purine nucleotides in an INT3 motif = 4.702/6, minimum = 2/6, maximum = 6/6) (Xu et al. 1993; Dirksen et al. 1994; Tanaka et al. 1994; Gersappe and Pintel 1999; Fairbrother et al. 2002; Caceres and Hurst 2013). As stop codons are also purine rich (6/9 nucleotides in the stop codons are purines), the INT3 motifs should be more conducive to including stop codons. Thus, distorted purine content within both ESEs and stop codons is unlikely to explain why stop codons are, in absolute terms, underemployed in ESE motifs. Nonetheless, we can ask whether after controlling for purine content the stop codons are specifically underemployed as our transfer selection model predicts.

To examine this, we identified the 6,856 tricodon sets that exactly match the purine content of the stop codon set (fig. 1*C*). The majority of these purine-matched sets (5,497/6,856 = 80.18%) have a higher FE than for the stop codon set (*P* < 2.2 × 10^−16^, one-tailed exact binomial test, null probability of success = 0.5). This implies the stop codon depletion in ESEs is specific to stop codons and not explained by purine content. Neither this result nor that for GC-matched sets above can be explained by allowing stop codons to exist in the matched codon sets or by the inability for stop codons to overlap one another (supplementary text 1, Supplementary Material online).

We can also control for both parameters simultaneously by considering tricodon sets that have both GC and purine content exactly matching the stop codon set (*N* = 413, fig. 1*C*) (e.g., the set {AAA, AAC, ACT}). We find that significantly more of these GC-purine-matched codon sets have greater FE than the stop codon set (317/413 = 67.02%, *P* = 5.484 × 10^−14^, one-tailed exact binomial test, null probability of success = 0.5, fig. 2*B*). In sum, we conclude that the depletion of stop codons in ESEs is relatively specific to the stop codons themselves, rather than being owing to the peculiarities in nucleotide content of ESEs and stop codons.

### The Stop Codon Depletion Is a General Property of ESE Motifs Defined within CDS

**FIg. 3.**
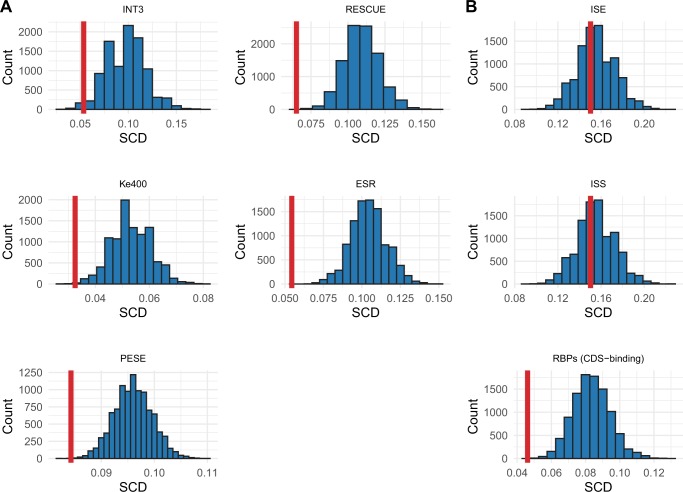
Histograms of the SCDs in 10,000 sets of dinucleotide-matched null pseudo-motif sets. The SCD in the real motifs of each set is shown by the vertical line. (*A*) Each ESE motif set demonstrates a significant stop codon depletion. (*B*) SCDs in the motifs of ISEs, ISSs, and CDS-binding RBPs. These depletions accord with their locations—intronic motifs not avoiding stop codons, CDS exonic motifs avoiding stop codons.

Another possibility that may explain the above depletion is a peculiarity of the motifs contained in INT3 set. To address this and ask whether the stop codon depletion applies to ESEs more generally, we calculated the FE score for the stop codon set in several ESE collections derived from analyses of coding exons. As expected, stop codons are significantly depleted in all ESE sets (table 1 and fig. 3*A*; note, the INT3 is not fully independent of the RESCUE-ESE, ESR, and Ke400 sets). This result also confirms that the INT3 set is representative of ESE sets more generally. Stop codons in the Ke400 set (Ke et al. 2011), unexpectedly enriched in exon cores and under positive selection (Caceres and Hurst 2013; Savisaar and Hurst 2018), are also significantly depleted (*P* ≈ 0.001, one-tailed empirical *P* value) consistent with depletions due to functioning within coding regions. These results also argue against the depletion in the INT3 set being a result of motif ascertainment biases resulting from the methods used to identify any particular set of ESEs (see “Motif sets” section for an overview of how each set was derived).

**Table 1.** SCDs and FE Scores Calculated from Dinucleotide-Matched Controls for Various RNA-Binding Protein Motif Sets.

Motif Set	Number of Motifs	Proportion Containing Stop Codons	SCD	FE	*P* Value^a^
INT3 ESE	84	0.107	0.054	−0.459	0.020
RESCUE-ESE	238	0.126	0.065	−0.404	9.999 × 10^−5^
Ke400 ESE	400	0.063	0.033	−0.391	0.001
ESR ESE	285	0.109	0.054	−0.479	9.999 × 10^−5^
PESE ESE*	2,069	0.222	0.084	−0.122	5.000 × 10^−4^
ISE^§^	110	0.436	0.150	−0.034	0.417
ISS^§^	103	0.427	0.146	−0.068	0.307
RBP motifs (CDS)	232	0.103	0.046	−0.450	9.999 × 10^−5^

NOte.—The PESE motif set marked * was derived from analysis of constitutively spliced noncoding exons, unspliced pseudoexons, and 5′ untranslated regions of intronless genes. The motif sets marked § indicate those not located within CDS.

a One-tailed empirical *P* value asking whether the real set of motifs have significantly less stop codons than simulated motif sets.

To avoid covariance with CDS parameters (such as codon usage), the PESE set (Zhang and Chasin 2004) was derived from comparisons of constitutively spliced noncoding exons, unspliced pseudoexons, and 5′ untranslated regions (UTRs) of intronless genes. Motifs in this set are therefore not subject to protein-coding constraints and should provide an exception to the rule. For this set, the SCD (0.084) is higher (*P* = 0.001, one-tailed one-sample *t*-test) and FE (−0.122) negative but higher (*P* = 0.008, one-tailed one-sample *t*-test) than for other ESE sets (table 1). This result is in the direction we expect and consistent with our model. That the FE is not 0 is likely a result of ESEs in this set also featuring in the ESE sets derived from CDS exons (Caceres and Hurst 2013), suggesting that some of these ESEs are likely functional in CDS and subject to protein-coding constraint.

### The Stop Codon Depletion Is a General Property of Motifs That Function in CDS

These results could, however, also be explained if there is a general avoidance of stop codons in all splice-related or RNA-binding protein (RBP) motifs whether they bind CDS or not. By contrast, our hypothesis predicts that motifs that do not function in CDS should not have a significant depletion. To ask whether the constraint is specific to motifs that do function in CDS, we consider FE for CDS-binding RBPs more generally and motifs associated with intronic binding.

The general set of RBP motifs thought to be CDS binding (Savisaar and Hurst 2017) (compiled from RBPDB [Cook et al. 2011], RBPmap [Paz et al. 2014], SFmap [Paz et al. 2010], and CISBP-RNA [Ray et al. 2013]) demonstrates a similar significant stop codon depletion (*P* ≈ 9.999 × 10^−5^, one-tailed empirical *P* value). For the non-CDS motifs, both intronic splice enhancers (ISEs) (*P* ≈ 0.417, one-tailed empirical *P* value) and intronic splice silencers (ISSs) (*P* ≈ 0.307, one-tailed empirical *P* value) have no avoidance of stop codons (fig. 3*B*). These results therefore argue that the depletion of stop codons in motifs functioning in exonic sequence is not ESE specific, splice specific nor a result of being an RBP-binding motif, but rather a peculiarity associated with being located in exonic CDS. Further, we find no evidence that stop codons containing ESE motifs are avoided in protein-coding sequences and cannot be discounted as being suboptimal (supplementary texts 2–5, Supplementary Material online).

### Multiexon lincRNA Sequences Are Significantly Depleted in Stop Codons

Does the lack of stop codons within ESEs transfer to and constrain lincRNA sequences as we propose? To test this, we employed the set of lincRNA sequences identified by Cabili et al. (2011). In this set, potential protein-coding transcripts were removed (see Materials and Methods for details) and so this set should contain a minimized number of lincRNAs with potential protein-coding ORFs that would contaminate our results. After our filtering, we employ 1,919 multiexon lincRNAs (53 from multigene families and 1,866 from singleton families, see Materials and Methods).

To eliminate the possible effects of nucleotide bias of the lincRNA sequences, we ask whether mature lincRNA transcripts are depleted for stop codons given the underlying nucleotide content of each sequence. We shuffled the nucleotides within every lincRNA and calculated the SCD for that iteration of 1,919 shuffled “pseudo-lincRNAs.” After repeating this for 1,000 iterations to generate a null distribution, we find no simulated iteration with overall SCD as low or lower than the SCD in the real 1,919 lincRNA sequences (true SCD = 0.130, FE = −0.162, *P* ≈ 9.99 × 10^−4^, one-tailed empirical *P* value, table 2). As the absence of a potential ORF was used to classify RNA species as lincRNA (rather than mRNA), this is a potentially conservative estimate.

**Table 2.** A Summary of Various Tests of Sequence Composition for Sequences in the Two lincRNA Data Sets.

	Sequence Set	
	Cabili et al. (2011)	Lagarde et al. (2017)
Number of sequences	1,919	456
SCD	0.130	0.128
FE^a^	−0.162*P* ≈ 9.99 × 10^−4^	−0.169*P* ≈ 9.99 × 10^−4^
Number of GC-matched codon sets with FE > stop codon set FE^b^	2,315/2,879 (80.41%)*P* < 2.2 × 10^−16^	2,300/2,879 (79.89%)*P* < 2.2 × 10^−16^
Number of GC-matched codon sets excluding stop codons with FE > stop codon set FE^b^	1,771/2,121 (83.50%)*P* < 2.2 × 10^−16^	1,751/2,121 (82.56%)*P* < 2.2 × 10^−16^
Number of sequences with exonic SCD < intronic SCD^b^	1,320/1,919 (68.79%)*P* < 2.2 × 10^−16^	325/456 (71.27%)*P* < 2.2 × 10^−16^
Median single-exon sequence exon SCD	n/a	0.139^c^
Median multiexon sequence exon SCD	n/a	0.122^c^
Median single-exon sequence exon FE	n/a	−0.148^d^
Median multiexon sequence exon FE	n/a	−0.162^d^

a One-tailed empirical *P* value.

b One-tailed binomial *P* value, null probability of success = 0.5.

c *P* = 8.878 × 10^−14^, Wilcoxon rank sum test between the SCD of each gene’s exons and introns.

d *P* = 8.878 × 10^−14^, Wilcoxon rank sum test between the FE scores of each gene’s exons and introns.

To confirm that this low SCD in lincRNAs is specific to the stop codons and not the GC content of the stop codons, we consider densities of GC-matched tricodon sets within the real and simulated sets of lincRNA sequences (converting codon set SCD values to FE values). The FE of the stop codon set is significantly lower than the FE of the majority of GC-matched control tricodon sets both when stop codons are permitted in the GC-matched codon sets (codon sets with FE > stop codon set FE = 2,315/2,879 = 80.41%, *P* < 2.2 × 10^−16^, one-tailed exact binomial test, null probability of success = 0.5, table 2) and when they are excluded (codon sets with FE > stop codon set FE = 1,771/2,121 = 83.50%, *P* < 2.2 × 10^−16^, one-tailed exact binomial test, null probability of success = 0.5). We conclude that the low SCD in lincRNA cannot be explained by the low GC content of the stop codons.

We find this stop codon depletion is also robust to pairwise analysis (i.e., each gene vs. randomizations of that same gene) (table 3), with 79.62% (1,528/1,919) of sequences having FE < 0 (*P* ≈ 0, one-tailed exact binomial test, null probability of success = 0.5). Of these, 493/1,919 have a significant depletion (*P* = 1.23 × 10^−200^, one-tailed exact binomial test, null probability of success = 0.05). Results are not affected by the choice of sequences from paralogous families (supplementary text 6, Supplementary Material online). Results are also quantitatively similar using a second independent set of sequences (GENCODE RNA Capture Long Seq annotated sequences, Lagarde et al. [2017]) (supplementary text 7, Supplementary Material online, and table 3). This trend is unlikely to result from hidden ORF contamination as the sequences 5′ of the most 5′ ATG, and therefore lacking protein-coding potential, also have reduced SCD (supplementary text 8, Supplementary Material online).

**Table 3.** A Summary of Individual Sequence FE Scores after Comparisons with Randomized Simulations of the Same Gene.

	Sequence Set	
	Cabili et al. (2011)	Lagarde et al. (2017)
Sequences with FE < 0^a^	1,528 (79.62%)*P* ≈ 0	416 (91.23%)*P* ≈ 0
Sequences with FE < 0, empirical *P* < 0.05^b^	493 (25.69%)*P* = 1.23 × 10^−200^	206 (45.18%)*P* = 2.33 × 10^−139^

a One-tailed binomial *P* value, null probability of success = 0.5.

b One-tailed binomial *P* value, null probability of success = 0.05.

Is this depletion specific to exonic lincRNA sequence as predicted by our transfer selection model? We compared the SCD in exons and introns of lincRNA sequences in a pairwise manner. This test is potentially conservative as some “intronic” sequence may well be hidden exon derived from unannotated alternative splice forms. However, we find that in 68.79% (1,320/1,919) of genes, the SCD of the exons is less than the SCD of the introns (*P* < 2.2 × 10^−16^, one-tailed exact binomial test, null probability of success = 0.5, table 2). Thus, the depletion appears to be more specific to exonic sequences, consistent with our model.

### Exons of Multiexon lincRNAs Demonstrate Significantly Reduced SCDs When Compared with Single-Exon lincRNA Exons

The above results are all consistent with our model of transfer selection. If we are to attribute this depletion to the presence of ESEs, the magnitude of the depletion in exons of single-exon lincRNAs should not be as great as that for multiexon lincRNAs, assuming single-exon genes do not need to contain ESEs to bind splicing factors. As the filtered Cabili et al. (2011) data set contained only 12 single-exon sequences in total, we performed this analysis on the GENCODE lincRNA sequences (Lagarde et al. 2017).

As expected, the SCDs of single-exon sequence exons (*N* = 877 exons) are significantly higher than the SCDs of the exons of multiexon sequences (*N* = 1,417 exons, N = 456 sequences) (median single-exon SCD = 0.139, median multiexon SCD = 0.122, *P* = 8.878 × 10^−14^, Wilcoxon rank sum test). However, given the compositional difference between single-exon and multiexon transcripts (median single-exon GC = 0.456, median multiexon GC = 0.477, *P* = 4.938 × 10^−12^, Wilcoxon rank sum test), it is important to control for the compositional differences of the exons for each class. We therefore calculated FE scores of single-exon sequence exons and multiexon sequence exons by simulating each exon sequence individually. Consistent with the above result and our expectations, FE scores for single-exon lincRNA are negative but significantly higher than for multiexon lincRNA (median single-exon FE = −0.148, median multiexon FE = −0.167, *P* = 0.027, one-tailed Wilcoxon rank sum test). That the single-exon genes also have a negative FE is not unexpected, as they are likely to be frequently bound by RBPs that also bind in CDS and contain ESEs that have splice-independent roles (Savisaar and Hurst 2016). In accord with the reduced SCD in single-exon lincRNAs, we also find a lower ESE density (median single-exon sequence exon ESE density = 0.127, median multiexon sequence ESE density = 0.155, *P* < 2.2 × 10^−16^, one-tailed Wilcoxon rank sum test). Confirming that the FE metric controls for GC differences, the slope on the line of FE predicted by GC is not significantly different from 0 (*P* = 0.334).

All else being equal, 5′ UTRs of protein-coding genes should have a lower SCD in single-exon transcripts than in multiexon ones, not least because the first intron is often close to the ATG and hence to the UTR. We find that there is a lower SCD in single-exon protein-coding genes than in multiexon protein-coding genes, although this is not robust to nucleotide control (see supplementary text 9, Supplementary Material online). For reasons unknown, the 5′ UTRs of single-exon protein-coding genes have higher ESE densities than for those of multiexon genes (see supplementary text 9, Supplementary Material online), which both runs counter to a priori expectations and conflates the above test.

### SCD Is Lowest in Regions Where ESE Density Is Highest

Although the above is consistent with reduced SCD in lincRNAs (compared with a nucleotide controlled null) as we predict, can we attribute this to ESEs and hence argue that the depletion is a result of CDS-imposed constraints on ESEs? If so, we expect SCD to be lowest in the regions in which ESEs typically reside. Despite selection on ESEs in protein-coding genes being most pronounced at exon ends (Berget 1995; Fairbrother et al. 2002; Fairbrother, Holste, et al. 2004; Carlini and Genut 2006; Parmley et al. 2006, 2007; Caceres and Hurst 2013), in lincRNA the proportion of sequence within 70 bp of an exon junction is not significantly correlated with evolutionary rate (Schuler et al. 2014), probably because in lincRNA ESEs function at the 5′ end more profoundly than at the 3′ end (Krchnakova et al. 2019). The depletion of stop codons and enrichment of ESEs should therefore be strongest at the 5′ end of lincRNA exons.

**FIg. 4.**
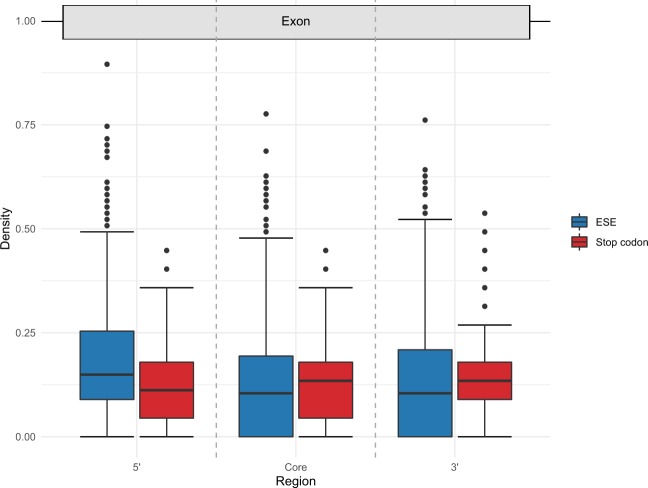
Densities of ESEs and stop codons in separate regions of lincRNA exon sequences longer than 207 nucleotides. 5′ flanks contain nucleotides 3–69 and 3′ flanks the corresponding nucleotides at the other exon terminus. Core regions are the 67 nucleotides centerd about the exon midpoint. In the 5′ flank region with higher ESE density, the SCD is reduced. In both the core and 3′ flank where ESE density is much reduced, SCD is increased. These trends are consistent with the presence of ESEs reducing SCD.

For each lincRNA gene, we divided each exon longer than 207 nucleotides into the 5′ flank (nucleotides 3–69), the equivalent 3′ flank and exon core (67 nucleotides centered about the exon midpoint), such that each region from each exon contained 67 nucleotides. We then calculated both ESE density and SCD for each region within each exon. As predicted, ESEs are enriched in 5′ flanking regions, whereas SCDs in this region are closer to 0 than either the core or 3′ regions (fig. 4). In accord with the notion that 3′ ends are not such key SR protein interaction domains, ESE densities in 3′ flanks are lower than in 5′ flanks and have higher SCDs. Similarly, exon cores have lower ESE densities and higher SCDs than 5′ flanks.

SCDs in the various regions differ significantly from null expectation (*χ*^2^ = 160.822, *P* = 1.20 × 10^−35^, chi-square test), with the 5′ region observed/expected frequency (O/E) lowest of all regions (5′ flank O/E = 0.917, core O/E = 0.960, and 3′ flank O/E = 1.1253). Further, when simulating each region separately, the stop codon FE for the 5′ flank (−0.185) is more negative than both the core (−0.142) and 3′ flank (−0.121) (all FE scores with empirical *P* values < 0.05). These broad-scale data are therefore consistent the lowest SCD being in the region where ESEs are most frequent.

### Reduced SCDs in lincRNA Sequences Are Attributable to the Presence of Predicted ESE Motifs

Can we attribute the depleted SCD in lincRNAs to ESEs directly? We compiled a consensus set of motifs from the nonredundant union of all ESE motif sets (2,582 motifs, 468 hexamers, and 2,060 octamers), excluding the Ke400 set as motifs in this set demonstrate positive selection and enrichment in exon cores over flanks (Caceres and Hurst 2013; Savisaar and Hurst 2018) despite splice mutations being enriched at exon ends (Woolfe et al. 2010). By excluding any sequence that matches a motif within the consensus set after predicting hits to all motifs to recover overlapping motifs, the influence of ESEs on SCD is eliminated. If ESEs are driving the depletion, the remaining (unmatched) sequence should have SCD similar to that predicted by its underlying nucleotide content.

We predicted hits to the consensus ESE motifs in each lincRNA and retained only the unmatched sequence. After randomly shuffling the remaining nucleotides, we observe that the real non-ESE sequence has a higher SCD than null (FE = 0.159, *P* ≈ 9.99 × 10^−4^, one-tailed empirical *P* value, supplementary table 1, Supplementary Material online) indicating the overall depletion of stop codons is owing to ESE motifs. This result further argues against the net depletion of stop codons in lincRNAs being an artifact of hidden protein-coding ORFs, as such a model predicts stop codon depletion both within and outside ESEs. Why the remaining sequence is enriched in stop codons is unknown, but could be the result of selection on the remaining non-ESE sequence to “appear” less like ESE to SR proteins to prevent inappropriate binding (e.g., see Savisaar and Hurst 2017) (supplementary text 10, Supplementary Material online). We also find that the depletion of stop codons is not a result of lincRNA sequences avoiding the use of those ESE motifs that contain stop codons (supplementary texts 11 and 12, Supplementary Material online).

### Skewed Stop Codon Usage in ESEs Reflects Skewed Stop Codon Usage in lincRNA

Above we have treated the stop codons as a single set. However, in all ESE sets (including INT3 and the consensus set), TGA is more abundant than TAA or TAG (table 4). This provides us with a further test of our transfer selection model. If the stop codon avoidance in lincRNAs is owing to ESEs avoiding stop codons, the avoidance of TAA and TAG in ESEs should be reflected in the usage of each stop codon within lincRNAs.

**Table 4.** Density of Each the Three Stop Codons in Each of the ESE Motif Sets.

Motif Set	Codon Density in Motif Set		
	TAA	TAG	TGA
INT3	0	0	0.054
ESR	0.005	0.011	0.039
Ke400	0	0	0.033
PESE	0.005	0.007	0.071
RESCUE	0	0	0.065
Combined	0.005	0.007	0.068

Using the Cabili et al. (2011) set of lincRNAs, we find that the stop codons in lincRNA are not used at similar frequencies, with TGA the most abundant (TAA density = 0.043, TAG density = 0.027, and TGA density = 0.060). When compared with null randomized shuffled lincRNA sequences, both TAA and TAG are significantly depleted (TAA: FE = −0.292, *P* ≈ 0.001; TAG: FE = −0.439, *P* ≈ 0.001, one-tailed empirical *P* values), whereas TGA is significantly enriched (FE = 0.247, *P* ≈ 0.001, one-tailed empirical *P* value). Thus, the stop codons most avoided in ESEs are those most avoided in lincRNA.

To attribute this directly to the presence of ESE motifs, we also ask whether the significant depletion of TAA and TAG occurs when ESEs are not present. If the TAA and TAG depletions remain in non-ESE sequence, this would argue for depletion due to reasons other than ESEs. As before we considered the lincRNA sequence that remains after the removal of sequence matching motifs in the consensus ESE set. After removal, both TAA and TAG are now found significantly more frequently than expected (FE = 0.590, *P* ≈ 0.001 and FE = 0.117, *P* ≈ 0.001, respectively, one-tailed empirical *P* values), whereas TGA is depleted (FE = −0.164, *P* ≈ 0.001, one-tailed empirical *P* value). We conclude that the depletion of both TAA and TAG in ESEs appears to force lincRNA sequences to also underemploy these two stop codons, consistent with our model.

### The Majority of lincRNAs Contain Permissible Pseudo-ORFs Longer than Expected by Chance

Taken together the above results are consistent with our model, transfer selection forcing a low density of stop codons in lincRNAs. Might this impact gene annotation? To distinguish noncoding RNA from protein-coding sequence, computational annotation approaches often consider the lengths of potential ORFs (Frith, Bailey, et al. 2006; Clamp et al. 2007; Dinger et al. 2008). To reduce the likelihood of falsely categorizing noncoding RNAs, putative noncoding RNAs are considered as those lacking ORFs longer than 300 bp as the majority (>95%) of annotated eukaryotic proteins are thought to be longer than 100 amino acids (Frith, Bailey, et al. 2006; Clamp et al. 2007; Dinger et al. 2008).

Our results above, however, have implications for any potential lincRNA pseudo-“ORF” (pORF) lengths. If the net depletion of stop codons constrains lincRNA sequences as we suggest, lengths of potentially tolerated lincRNA pORFs should be longer than expected. Indeed, Niazi and Valadkhan (2012) show a nonnegligible proportion of “functional” long noncoding RNAs (lncRNAs), although not intergenic, have an “ORF” length > 300 nucleotides (e.g., the *Xist* gene encodes a functional ≈15-kb transcript in mouse [Prasanth and Spector 2007] with a potential 592 nucleotide ORF [Brockdorff et al. 1992]).

To address the likely extent to which true noncoding lincRNAs present long pORFs by chance, we generated 1,000 sets of simulated lincRNA sequences by shuffling the full multiexon lincRNA transcripts. For each real and simulant sequence, we determined the length of the longest pORF, assuming pORFs start ATG and terminate with a stop codon in the same reading frame. Seven real sequences had no complete pORF in any frame and were excluded. We calculated the *Z* score for each sequence, with a positive *Z* indicating an increased maximum pORF length compared with null sequences.

**FIg. 5.**
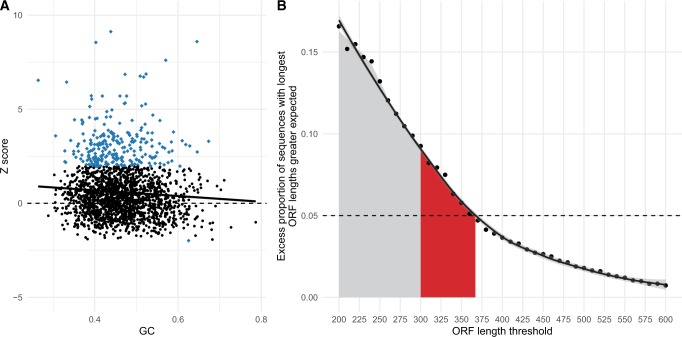
Analyses of potential ORFs in lincRNA sequences. (*A*) *Z* scores for the longest permissible ORF lengths when compared with randomly shuffled simulated sequences are negatively correlated with GC content (*ρ* = −0.082, *P* = 2.976 × 10^−4^, Spearman’s rank correlation). Moreover, data points demonstrating significant deviations (blue diamond) from expected ORF are all positive, except one. One sequence with *Z* = 32.519 has been removed from the figure for visual purposes. (*B*) The excess proportion of sequences with maximum ORF lengths longer than expected decreases with increasing ORF length thresholds. A threshold of 368 bp is required such that there is less than a 5% excess (dotted line). Results for thresholds within the region highlighted in red demonstrate areas of ORF lengths that could be ambiguous if used as the sole determinant of coding capability, extending beyond thresholds that are up to and including the commonly used 300-bp threshold (gray).

We find robust evidence that pORFs are commonly longer than expected, with 62.33% (1,227/1,912) having *Z* > 0 (*P* < 2.2 × 10^−16^, one-tailed exact binomial test, null probability of success = 0.5, median longest pORF length: real = 159, simulants = 129; maximum longest pORF length: real = 2,202, simulants = 450). Further, more sequences than expected by chance also have a significantly positive *Z* (13.13% = 251/1,912, *P* < 2.2 × 10^−16^, one-tailed exact binomial test, null probability of success = 0.05). Only one sequence had a significantly shorter pORF than expected (*P* ≈ 1, one-tailed exact binomial test, null probability of success = 0.05). These differences in pORF length are greatest when AT-content is highest, that is, when stop codons are more likely to occur by chance (*ρ* = −0.083, *P* = 3.00 × 10^−4^, Spearman’s rank correlation between sequence GC content and sequence pORF length, fig. 5*A*). Thus, it would appear that not only are permissible pORFs longer than expected, but there exists greater deviation from expected pORF lengths (measured in standard deviation units) when stop codons should be more frequent.

### Almost 10% of Sequences Would be Misannotated If Categorized on ORF Length Alone

Do longer than expected pORFs have implications for lincRNA sequence identification? Although the 300-bp ORF lower limit is applied to reduce false-positive rates (Frith, Bailey, et al. 2006; Clamp et al. 2007; Dinger et al. 2008), sequences are also annotated based on their level of sequence conservation as noncoding RNAs demonstrate conservation but below that of protein-coding genes. However, a conservation approach is a priori poor at identifying young ORFs. Given pORF lengths are increased owing to stop codon avoidance, we ask what a safe length threshold might be.

We find 11.57% (222/1,919) of the total sequences meet or exceed the 300-bp threshold in our data (taking the median length for sequences grouped into gene families). However, is this number biologically relevant? For example, random sequences of equal length to lincRNA sequences may also contain pORFs longer than 300 bp. To test this, we concatenated all exons from all sequences, randomly shuffled the concatenation and extracted randomized sequences with lengths matching the real mature transcript sequences, thereby generating 1,000 randomized null sets of sequences with equal overall transcript length and nucleotide content. For each iteration of randomized sequences, we then calculated the number of sequences with a pORF exceeding 300 bp. We find the number of real lincRNA sequences exceeding the threshold (222) is almost significantly greater for the null sets (mean number exceeding = 44.434, standard deviation = 171.770, *P* ≈ 0.051, one-tailed empirical *P* value). Further, no randomized set had a pORF longer than the longest pORF seen in the true lincRNAs (*P* ≈ 9.99 × 10^−4^, one-tailed empirical *P* value, maximum true = 2,202, maximum simulant = 594). Using the mean number of simulant sequences exceeding the 300 bp threshold as the expected number to exceed the threshold, this suggests $\frac{222-44.434}{1,919}\;\approx 9.25\%$ of real sequences could be misclassified based on ORF length alone beyond that expected by chance.

The above results suggest that owing to transfer selection, achieving a 5% false-positive rate requires a threshold longer than 300 bp. How long might this cutoff be? Using ten nucleotide threshold intervals between 200 and 600, we calculated percentage excesses over null as above and fitted a local regression model. This model predicts a threshold of 368 bp is required so that only 5% of sequences exceed the threshold (fig. 5*B*). However, although a longer threshold reduces the false-positive rate, we note that there likely exists an abundance of functional protein-coding genes that encode short proteins (Oyama et al. 2004; Frith, Forrest, et al. 2006; Andrews and Rothnagel 2014; Slavoff et al. 2014). Thus, a longer threshold will also increase the false-negative rate. Given this, bioinformatic approaches should be coupled with experimental validation (Kashi et al. 2016) whenever possible.

## Discussion

Although much is known about the selective pressures acting on CDSs, those in noncoding sequences are less well understood. Human lincRNAs are under weaker purifying selection than protein-coding genes (Marques and Ponting 2009; Cabili et al. 2011; Haerty and Ponting 2013) and contain fewer conserved regions (Pang et al. 2006). However, ESE motifs that are under strong purifying selection in protein-coding genes (Parmley and Hurst 2007; Parmley et al. 2007; Warnecke et al. 2008; Smithers et al. 2015; Savisaar and Hurst 2018) are also under purifying selection in lincRNA sequences, suggesting splicing of multiexonic lincRNA transcripts is also important for function (Schuler et al. 2014; Haerty and Ponting 2015). With both coding and noncoding sequence thought to undergo the same splicing process by the same splice machinery (Will and Luhrmann 2011; De Conti et al. 2013; Krchnakova et al. 2019), we hypothesized that the same constraints should apply to both types of sequence.

Here, we have provided evidence consistent with a depletion of stop codons found in ESE motifs that, allowing for nucleotide content, is specific to the stop codons. That both ESEs and stop codons are purine rich makes the depletion of stop codons particularly noteworthy (indeed the high purine content may be a defining feature of ESEs to discriminate exon ends from other sequences, see supplementary text 13, Supplementary Material online). The evidence that we have presented suggests that this stop codon depletion of ESEs that function in CDS transfers to lincRNA sequences. As a consequence, and contrary to null expectations, lincRNAs too are significantly depleted in stop codons. Multiple lines of evidence, including a significant increase in stop codons found after removing ESEs from lincRNA sequences, suggests that ESEs are the origin of this depletion (or at least a major contributor). Thus, constraints imposed on motif composition in protein-coding sequences can transfer to noncoding sequence.

One could argue that the most obvious alternative explanation for the depletion of stop codons in lincRNA is the contamination of the data set with true, but unrecognized, protein-coding sequences. However, several pieces of evidence argue against this. First, if a lack of ORF is used to classify RNA species as lincRNA, rather than mRNA, we expect an enrichment of stop codons in lincRNA. Our tests comparing SCDs are thus conservative. Second, we observe similar depletions from two independent data sets, in which both take measures to exclude sequences demonstrating evidence of protein-coding potential. That there is also a depletion of stop codons in exon regions with the highest ESE density, yet no depletion in exonic sequence after the removal of ESEs (and indeed an enrichment), suggests lincRNA sequences are not depauperate in stop codons in their entirety but biased by the presence of ESEs. Furthermore, the sequence upstream of the first ATG is stop codon depleted, despite no influence of any ORF on densities (supplementary text 8, Supplementary Material online).

We also question whether ORF contamination could explain the magnitude of the observed reduction in SCD. Any contamination by real hidden protein-coding ORFs would also have to be substantial, particularly given the pairwise analysis of SCDs against randomizations of each gene indicates that 79.62% of sequences have a stop codon depletion. Given the filters on the original sequences, it seems unlikely that true ORFs, common enough to provide such contamination, would have gone unrecognized.

In principle, lincRNA sequences may be depleted in stop codons if they overlap unannotated protein-coding genes on the same strand. Unless there is a rich source of unannotated overlapping ORFs this is not parsimonious to explain the commonality of stop codon depletion. Moreover, in neither the hidden ORF nor the unannotated overlapping ORF model is the specificity of stop codon depletion to ESEs and exon 5′ ends (where ESEs are most abundant) explained. That the stop codons depleted in lincRNA (TAA and TAG) accord with the stop codons depleted in ESEs also supports transfer selection above ORF contamination. In sum, transfer selection therefore provides the most parsimonious explanation of our observations.

We have also assumed that as SR proteins must bind coding exons there is a constraint transferred to noncoding exons. Might there be a transfer in the opposite direction? If we consider CDS alone, a theoretical set of motifs with most utility (most likely to hit exons exclusively) would be one that avoids stop codons entirely. Over evolutionary time, selection might therefore be expected to eliminate ESEs with stop codons as potential binding motifs. Yet stop codon containing motifs persist. However, RBPs and binding motifs are thought to coevolve which has been exploited to predict RBP-binding domains (Yang et al. 2018). If these stop codon containing motifs can be more easily employed in noncoding sequence while also providing the adequate binding capability, then they might still provide enough splicing functionality to be selected for. Given only a minority of transcribed sequence is protein-coding (Encode Project Consortium 2012), the relative frequency of noncoding RNA splicing may render such motifs selectable. In turn, they may also then be useful motifs in protein-coding sequence where splice specificity is less important, but the nucleotide composition of the sequence allows their usage. Thus, if motifs that include stop codons are of utility and can be frequently used within lincRNA, it may be that RBPs and stop codon containing motifs coevolve such that they persist as functioning motifs. A suggestion of this is found in our result that the ESEs that feature stop codons are if anything overused on a per motif basis in CDS (supplementary text 2, Supplementary Material online).

### Stop Codon Depletions and the Origin of De Novo Genes

The stop codon depletion in lincRNA and ESEs more generally might modulate the evolution of new genes. The origin of new genes receives much attention (overviews in Long et al. 2003; Kaessmann 2010; Tautz and Domazet-Loso 2011; McLysaght and Hurst 2016). Although duplication and rearrangement (Ohno 1970; Jacob 1977; Zhang 2003; Ciccarelli et al. 2005; Innan and Kondrashov 2010; Magadum et al. 2013; Van Oss and Carvunis 2019) are known to be important processes that adapt and reuse functional sequence, the creation of de novo protein-coding genes from previously nonfunctional or noncoding sequences is increasingly being recognized as a source of novelty (Tautz and Domazet-Loso 2011; McLysaght and Guerzoni 2015; McLysaght and Hurst 2016).

Two important steps are required to give rise to and allow fixation of functional proteins from noncoding sequence: acquisition of uninterrupted ORFs and regulatory transcriptional signals. The order of these events is not clear nor necessarily uniform, with two models proposed each arguing for the respective events occurring first (McLysaght and Guerzoni 2015; Schlotterer 2015). In the “RNA-first” scenario, the abundance of lncRNAs that are transcribed and, possibly accidentally, associated with ribosomes (Wilson and Masel 2011; Ruiz-Orera et al. 2015) makes it possible that many unintended peptides are actively translated, thereby becoming protogenes. In an “ORF-first” scenario, if an ORF is already present within the sequence mutations in *cis* regions could induce expression of the ORF (Kaessmann 2010; Zhao et al. 2014).

LincRNAs containing longer than expected ORFs owing to stop codon depletion are relevant to the RNA-first model. What is unknown is how the length of the pORF of a protogene relates to the probability of evolving from proto to functional protein-coding gene. If longer sequences are more likely to find immediate utility, rather than be toxic (Boyer et al. 2004; Levine et al. 2006), then this should exaggerate any putative tendency for de novo genes to originate in GC-rich sequence. Although ORF lengths in AT-rich regions have a greater deviation from expected (fig. 5*A*), the raw ORF lengths are longer in GC-rich domains (correlations between GC raw ORF lengths are significantly positive, *ρ* **=** 0.219, *P* < 2.2 × 10^−16^, Spearman’s rank correlation). Further, GC-rich regions are more transcriptionally active (Lercher et al. 2003) with transcription factor binding sites being GC rich (Wang, Zhuang, et al. 2012), and therefore more likely to give rise to lincRNA expression.

### Stop Codon Avoidance Is Seen for Other RBP Motifs

Although above we have considered ESEs and show that they contain few stop codons, in principle, these are only one exemplar of CDS exonic motifs subject to stop codon depletion and hence subject to transfer selection. An expectation of stop codon depletion should then not be limited to ESEs but should also apply to other RBP-binding motifs that function within coding regions. We indeed find a broader set of such motifs (compiled by Savisaar and Hurst [2017]) has a significant depletion of stop codons (*P* ≈ 9.999 × 10^−5^, one-tailed empirical *P* value, table 1 and fig. 3). We caution that only conservative conclusions should be drawn from this result as the quality of motifs used in the set is thought to vary (Savisaar and Hurst 2017). Nonetheless, we suggest that any peculiarities of sequence content necessitated by binding within CDS could have multiple transfer modes. It remains to be seen to what extent the compositional properties of lincRNAs are a consequence of carryover of binding preferences of RBPs shared with CDSs.

It is also the case that transfer selection should not be considered restricted to RBPs but may apply in other contexts and not limited to stop codons. Here, we consider the comparison between coding and noncoding sequence, yet in theory similar logic could be applied to anything that interacts with two different sequence types. For example, there may be proteins that interact within different regions at the DNA level and transfer constraints between them.

## Materials and Methods

### General

Analyses were conducted using custom Python 3.6.4 scripts (available at https://github.com/la466/lincrna_stops_repo) using standard, readily available Python libraries. R version 3.5.1 (R Core Team 2018) was used for statistical testing and plotting of figures. BEDTools version 2.27.1 (Quinlan and Hall 2010) was used for operations performed on sequence coordinate data. For motif simulations, 10,000 iterations were run. For all other simulations, 1,000 iterations were run unless specified.

### Retrieval and Filtering of lincRNA Sequences

LincRNA sequence coordinates were downloaded from the supplementary data set 2, Supplementary Material online, “TraitTable” sheet of Cabili et al. (2011). Sequences identified by Cabili et al. (2011) were done so via four key steps: 1) transcriptome reconstruction from RNA-seq data using two transcript assemblers (Cufflinks and Scripture), 2) compilation of all noncoding and unclassified transcripts previously annotated, 3) determination of unique isoforms from each transcript locus by integrating RNA-seq reconstructions with all annotation resources (Cuffcompare), and 4) processing of transcripts to identify those reliably expressed, large, multiexonic, noncoding, and intergenic. Of these, the lowly expressed transcripts were removed using a learned read coverage threshold. Noncoding transcripts were filtered from novel potential protein-coding transcripts by removing those with evolutionary constraint to preserve amino acid content in any of the three reading frames (those with a positive phylogenetic codon substitution frequency metric [Lin et al. 2011]) and by excluding transcripts matching a protein-coding domain present in the Pfam database (Finn et al. 2010).

From the supplementary data, Supplementary Material online, only entries with the “ConservativeSet” flag set to 1 were retained, to leave 4,662 data points. These sequences are those with no evidence of protein-coding potential and that can be reconstructed in at least two different tissues or reconstructed by two assemblers in the same tissue. As such, transcripts with insufficient coverage should also have been removed. This sequence set should therefore contain a minimized number of potential protein-coding transcripts. Sequences containing noncanonical nucleotides and those containing only one exon were removed, leaving 4,646 multiexon sequence data points.

To limit the effects of retaining genes with similar composition from our results, genes were clustered into paralogous families. The sequences were BLASTed all against all (nucleotide–nucleotide BLAST 2.4.0+ [Camacho et al. 2009]). Starting with a randomly selected sequence, all sequences that had a significant hit were grouped as part of the same family and considered a single data point for the analyses. After grouping into paralogous families, 1,919 data points remained. For analyses, either the median value for sequences that are members of the same family was taken or one member selected at random to represent the family. Where one member was selected at random, the analysis was repeated multiple times to avoid biases resulting from the random family member chosen.

Intergenic GENCODE lncRNA sequences reannotated by RNA Capture Long Seq from heart, testes, liver, brain, human K562, and human HeLa cells were also used (Lagarde et al. 2017). Sequence IDs corresponding strictly and exclusively to lncRNA were obtained from Lagarde et al. (2017, supplementary data set 1) (although annotated as lncRNA, these sequences are intergenic and therefore appropriate). A processed bed file containing only entries for full-length transcripts whose 5′ end is supported by FANTOM5 CAGE transcription start site data and 3′ is polyadenylated (cage + polyASupported) was downloaded from the GEO database accession GSE93848 (last accessed May 24, 2019). Only entries corresponding to the exclusive lncRNA IDs were retained. From these, the full-length multiexonic transcripts containing only canonical nucleotides were built, retaining only those longer than 200 nucleotides to leave 11,083 transcript sequences. These were then subject to clustering into paralogous families as before, leaving 456 multiexon data points for analyses. No sequences were identical to sequences from Cabili et al. (2011). The exons of single-exon lincRNAs were also extracted (*N* = 2,972) and clustered into paralogous families, leaving 877 single-exon data points.

### Retrieval and Filtering of Protein-Coding Sequences

Protein-coding sequences were retrieved using similar protocols to Savisaar and Hurst (2016). To extract genome features, both the genome sequence and genome features were downloaded from the Ensembl database (Zerbino et al., 2018; Release 94, ftp://ftp.ensembl.org/pub/release-94/, last accessed October 25, 2018). The genome features were queried and only those labeled as “CDS” and “protein-coding” were retained. From these features, the full CDS was constructed leaving 98,382 CDSs in the data set. This data set was filtered to remove CDSs that contained noncanonical bases, were not of a length divisible by 3, did not start with ATG, and did not end with a stop codon or contained in-frame stop codons. If more than one transcript per gene was present, the longest was retained; if two with the same length per gene were present, the first to be queried was retained.

The genome sequence and features for the *Macaca mulatta* genome were also obtained from the Ensembl database (Zerbino et al. 2018; Release 94, ftp://ftp.ensembl.org/pub/release-94/, last accessed November 5, 2018). Orthologs for all human genes remaining after the filtering steps described above were obtained via an Ensembl Biomart query using the Pybiomart Python package (https://github.com/jrderuiter/pybiomart, last accessed November 14, 2018). The orthologous CDSs of *Macaca* *mulatta* that corresponded to the remaining filtered human genes were extracted in the same process as for human CDSs and filtered according to the previous criteria. Both the human and macaque CDSs were translated to protein sequences and aligned using MUSCLE v3.8.31 (Edgar 2004) via the Biopython wrapper. Once aligned, the sequences were converted back to the corresponding DNA sequences. The *d*_S_ and *d*_N_/*d*_S_ scores of the human/macaque alignments were calculated using PAML codeml (Yang 2007) using the Bio.Pyhlo module (Talevich et al. 2012) from the Biopython wrapper, with the settings *seqtype = 1*, *runmode = 0*, *model = 0*, *Nsites = []* and an arbitrary tree. Only CDSs that produced a *d*_S_ score of >0.2 or a *d*_N_/*d*_S_ score of >0.5 were retained to minimize the risk of pseudogene contamination (Savisaar and Hurst 2016). After this filtering, 13,187 multiexon sequences remained.

Sequences were then grouped as before into paralogous families. Single-exon sequences (1,036) were also extracted and grouped into paralogous families. 5′ UTR sequences of both multi- and single-exon sequences were obtained by constructing the full-length mature transcript and querying for the index for where the CDS starts. The 5′ UTR was defined as all nucleotides up to this index point. Introns of the sequences were extracted from the genome sequence using the coordinates from the relevant exon entries.

### Motif Sets

The INT3 motif was downloaded from the supplement of Caceres and Hurst (2013). Other ESE motif sets except Ke400 were obtained as described in Caceres and Hurst (2013) and Savisaar and Hurst (2018). ISEs were obtained from the supplement of Wang, Ma, et al. (2012). ISS motifs were obtained from the supplement of Wang et al. (2013). RBP motifs were obtained from the supplement of Savisaar and Hurst (2017). For RBP motifs, those that had significant enrichment *P* values were considered CDS binding and those with significant depletion *P* values were considered non-CDS binding. We provide a brief overview of each ESE motif set below:

*RESCUE*. Motifs were derived computationally (Fairbrother et al. 2002; Fairbrother, Yeo, et al. 2004), on the assumption that ESEs should be enriched in constitutively spliced exons and avoided in flanking introns and be more frequently when splice sites are weak. Internal exons and flanking introns were queried. Results were experimentally validated and compared with prior data.

*Ke400*. A systematic experimental analysis (Ke et al. 2011) where all 4,096 hexamers were substituted at five positions in two internal exons in minigene constructs. These constructs were transfected to human cells with the splice promoting ability of each motif reported. The top 400 most potent splice modifying hexamers were retained for the Ke400 data set.

*ESR*. Motifs were derived computationally (Goren et al. 2006), searching human–mouse orthologous exons with the same lengths, shorter than 250 nucleotides and with classical GT-AG splice sites. Two expected metrics were used to query dicodon frequencies, assuming the two codons appear independently. The first, expected conservation rate, multiplied the probability of codon 1 to be conserved between human and mouse, the probability of codon 2 to be conserved between human and mouse, and the number of times the dicodon appeared conserved between human and mouse. For each dicodon, this reflects the expected frequency of observing a conserved human–mouse dicodon. The second, expected observation rate, multiplied the number of times the pair of amino acids encoded by the dicodon was detected in the data. These numbers were compared with the real frequency of conserved and occurred dicodons. Only dicodons that were statistically significantly overrepresented and highly conserved at synonymous sites were considered.

*PESE*. Computationally derived motifs (Zhang and Chasin 2004) comparing frequencies of octamers overrepresented in constitutively spliced noncoding exons versus unspliced pseudoexons and 5′ UTRs of intronless genes, assuming ESEs are not frequently in pseudoexons and UTRs are devoid of ESE activity. Experimental confirmation of many ESEs subsequently provided (Zhang et al. 2005).

*INT3*. The motifs that appear in at least three of the RESCUE, Ke400, ESR, and PESE data sets (Caceres and Hurst 2013). Considered a “gold-standard” set and designed to have a low false-positive rate.

### Generating Compositionally Matched Codon Sets

All permutations of three unique codons were generated (N = 64 × 63 × 62 = 249,984), including stop codons (fig. 1*A*). However, 3! = 6 permutations of the same three codons exist (e.g., the set {ATC, GAC, TCA} is equivalent to {GAC, TCA, ATC}) and so redundant sets were removed, leaving *N* = 249,984/6 = 41,664 codon sets (fig. 1*B*). To control for the net GC content of stop codons (fig. 1*C*), we filtered the remaining codon sets to retain only those with identical net GC content as the stop codon set, GC content of a codon set being defined as the sum of the number of G and C residues of the three codons divided by 9 (the number of nucleotides). For example, the tricodon set {AGT, AAT, GAT} has GC content of 0.222, the same as the stop codon set. There are 2,879 tricodon sets with net G and C content identical to the stop codon set.

A purine-matched subset (*N* = 6,856) was also derived by taking all sets with identical purine content as the set of stop codons (net purine content = 0.667). Note the size of the GC- and purine-matched codon sets differs as a result of the GC content (0.222) being more extreme than the purine content (0.666), with the smallest groupings of codon sets being those with the most extreme content, following binomial principles.

The intersection of these two groupings of tricodon sets contained those tricodon sets with both equal GC and purine content to the stop codons (*N* = 473). We performed further restrictions to generate sets with identical GC content but that contained no stop codons (*N* = 2,121) and with identical GC content but in which no codon could overlap with any others from the same set (*N* = 131).

### Generating Dinucleotide-Matched Motif Sets

Sequences within a motif set (e.g., INT3) were scanned for every dinucleotide in both reading frames (e.g., the motif GAAGTA contains the dinucleotides GA, AG, TA, AA, GT). The frequencies for each dinucleotide were totaled for all motifs in the data set. Then, for each simulation iteration, for each real motif (typically six nucleotides) a pseudo-motif of the same length was generated by randomly sampling dinucleotides with probabilities defined by the true dinucleotide frequencies calculated (i.e., for a real motif of length six, three dinucleotides were randomly sampled). If a motif was not of even length, a random nucleotide was sampled using the distribution of nucleotides in the true motif set and appended pseudo-motif. If the new pseudo-motif had already been generated in that iteration, it was removed and the process restarted. Each simulation iteration therefore contained an identical number of pseudo-motifs as the number in the true set.

### Density Calculations

We calculated density as outlined in the Introduction section. If a query motif overlapped another, overlapping nucleotides were only counted once. For example, for the query motif set {CCT, GGG} in the sequence TGATA**GGGG**A, we only consider the four nucleotides that match the query motifs.

Although we refer to this metric as “codon density” or “motif density,” this term can be slightly misleading as we count the number of nucleotides matching the motif/codon, not the number of matching motifs/codons per se. This density metric, however, does enable us to control for varying query motif or queried sequence lengths. The metric therefore describes how much of a particular sequence comprises by the query motifs (a codon, ESE, etc.) and therefore has a minimum of 0 (the sequence contains no nucleotides matching the query motifs) and maximum of 1 (all nucleotides in the sequences match one or more of the query motifs).

### Calculating FE Scores

We employ a FE metric in several cases to describe the deviations of true measures of abundance from that expected given underlying nucleotide distributions. Null expectations were obtained via iterated simulations. We provide an example below of calculating codon set density in ESE hexamers, but the same method is applied to calculating SCD in full-length lincRNA sequences, SCD in individual exons, and pORF lengths in the lincRNA sequences.

To calculate the FE of any given codon set in the INT3 ESEs, we first calculated the raw density of the codon set (as detailed above) within the true INT3 ESE hexamers. Second, having generated randomized sets of pseudo-ESE motifs (as detailed above; for other calculations, these are sets of randomized shuffled sequences), we calculated the density of the codon set in each iteration of the randomized pseudo-ESE motif sets. This provided us with a density score for the codon set in the real ESE motifs and distribution of density scores from the simulated motifs. FE was then calculated using the formula $\mathrm{FE}=\frac{O-E}{E}$, where *O* is the observed density of the codon set motifs in true motifs and *E* is the mean density of the codon set motifs in the simulant motifs.

FE as a metric has the benefit that FE < 0 implies a relative depletion given underlying nucleotide content, FE > 0 a relative enrichment, and FE ≈ 0 as expected given underlying nucleotide content.

### Calculating *Z* Scores

*Z* scores for pORF lengths were calculated similarly to FE scores. First, the longest pORF in any frame in the true sequence was calculated. Then, the longest pORF was calculated for each randomization of the lincRNA sequences. The *Z* score for each sequence was then defined as the real longest pORF length minus the mean of the group of simulated longest pORFs, divided by the standard deviation of the simulated longest pORFs, taking the median *Z* score for sequences that are members of a paralogous family.

### Predicting Hits to Motifs in Sequences

Regular expressions were used to predict hits to motifs in sequences using the standard built-in Python package. For each sequence, the indices for the hits to each motif were stored. Any subsequent hits to a motif were appended to this list. For each sequence, the list of indices was then filtered such that each index could only appear once. In this way, if two motifs overlapped, we would only consider the nucleotides that matched both only once in our calculations.

### Removal of Sequence Matching ESEs

To interrogate sequence that featured no ESEs given a particular ESE set, hits were first predicted to the ESEs for each query sequence. The index of each nucleotide hit that overlapped an ESE for each sequence was stored, and only once all motifs had been queried were these indices further considered. In this way, all overlapping motifs were identified. For each sequence, the positions corresponding to indices that were not stored were calculated and the corresponding sequence parts extracted. Sequence parts interrupted by a predicted ESE were treated as separate sequence parts. This prevents unexpected motifs being generated by concatenating the remaining sequence. For example, querying the sequence ACTAC**TTTTT**AGA for the motif TTT would have resulted in two unmatched parts, ACTAC and AGA. Analyses were then performed on these remaining sequences individually.

### Identifying Potential ORFs

Potential ORFs were identified by scanning each sequence for every ATG in every frame. For each ATG, downstream codons in the matching frame were then queried in order until a stop codon was identified. The nucleotide distance to the stop codon was stored and once all ATGs had been queried, the longest ORF was retained. Seven of the lincRNA sequences contained no potential ORF and so were excluded from the analysis.

### Calculating Empirical *P* Values

Empirical *P* values were calculated using outputs from the simulations using the formula $P\;\approx \;\frac{m+1}{n+1}$, where *m* is the total number of simulants scoring less than or equal to the real value and *n* is the total number of simulants. If the direction of the one-tailed test was in the opposite direction, *m* is the total number of simulants scoring greater than or equal to the real value.

## Supplementary Material

msz299-Supplementary_Data
