# Supplementary material for: A Depletion of Stop Codons in lincRNA is Owing to Transfer of Selective Constraint from Coding Sequences
Source: Mol Biol Evol. 2019 Dec 16;37(4):1148–64. doi: 10.1093/molbev/msz299 (PMC7086181; doi:10.1093/molbev/msz299)
Supplement: msz299-Supplementary_Data [file msz299-supplementary_data.zip › msz299-Suppl_Data/Supplementary Texts.pdf]

Supplementary Texts

Supplementary Text 1: The presence of stop codons within the tri-codon codon sets nor the inability for the stop codons to overlap can explain the stop codon depletion in the INT3 ESE motifs when compared with compositionally matched codon sets

We find that when controlling for both GC content and purine content, the stop codon set is depleted in the INT3 ESE motifs. However, are these results due to a subset of GC-matched codon sets also containing stop codons?

To eliminate this potential bias, we performed the same analysis as in the main text using tri-codon sets containing no stop codons and compared them with the stop codon set. N = 2,121 sets have identical GC content and contained none of the three stop codons. We find a similar result as before – 1,578/2,121 (74.40%) have higher FE than the stop codon set ($P < 2.2 \times 10^{-16}$, one-tailed exact binomial test, null probability of success = 0.5). When matching by purine-content, the removal of codon sets containing stop codons left N = 5,587 purine-matched codon sets. Again, 4,457/5,587 (81.56%) have higher FE than the stop codon set, a significant number ($P < 2.2 \times 10^{-16}$, one-tailed exact binomial test, null probability of success = 0.5). The high proportion of codon sets with a greater FE than for the stop codon set is therefore not a consequence of some stop codon sets containing stop codons.

It is also the case that no stop codon can overlap with another stop codon. If any given motif contains a stop codon, there is therefore less chance it will contain another stop codon. For example, in the motif G**TAA**AA, no second stop codon can exist without destroying the motif. Yet, if the query codon set is {AAA}, as AAA can overlap (e.g. GT**AAAA)** it is more likely to have a higher density. We therefore restricted the codon sets again, but only considered codon sets where no codon could overlap another (for example the sets {AAA, CCC, TAT} and {TTA, TTC, TTG}). For GC-matched codon sets with no overlapping codons (N = 131), 91/131 (69.47%) have greater FE than the stop codons ($P = 4.898 \times 10^{-6}$, one-tailed exact binomial test, null probability of success = 0.5). For the purine-matched non-overlapping sets (N = 712), 565/712 (79.35%) have a greater FE than for the stop codon set, again a significant number ($P < 2.2 \times 10^{-16}$, one-tailed exact binomial test, null probability of success = 0.5).

That some stop codons appear in the tri-codon sets and that stop codons are unable to overlap with themselves do not therefore explain their depletion in ESEs.

Supplementary Text 2: Stop codon containing ESEs are per motif more frequent in exonic sequence than ESEs that contain no stop codons, although differences can be attributed to differences in dinucleotide content

One possibility that might explain why stop codons are depleted in ESEs is that the motifs containing stop codons are for some reason poorer functioning ESEs in any context. Evidence in *Drosophila* and *C. elegans* suggests individual SR proteins have functional differences (Ring and Lis 1994; Hoffman and Lis 2000; Kawano et al. 2000; Longman et al. 2000; Kim et al. 2003) and by association, the motifs they bind may therefore be used at different frequencies. If so, there could be a selective pressure against using stop codon containing motifs. This may have a direct consequence in that they are used less frequently, but also an indirect consequence in that they are then less likely to be identified using computational approaches. In this scenario, there would be no reason to suppose any CDS-imposed constraint is transferred.

To address this, we ask whether ESEs that contain stop codons are suboptimal and hence underused given their relative frequency within the set of ESEs. If stop codon containing motif frequencies deviate from neutral expectations, it could indicate that selective pressures are acting against the usage of such motifs. We focus analyses on the "gold-standard" INT3 set of motifs of highly constrained core motifs likely to be true functional binding motifs.

If functional differences are leading to depletion, it is expected that the stop codon containing motifs are used less frequently per motif in exonic sequences. Hits to the INT3 motifs were predicted in human protein-coding coding exons, picking one transcript sequence per paralogous family at random (N = 6,045 family data points). The density of stop codon containing ESEs is much reduced (0.026) when compared with the density of the remaining ESEs (0.169). However, two factors could explain this difference. First, only 9/84 of INT3 motifs contain a stop codon. Second, stop codon containing ESEs cannot be incorporated into one of the three reading frames. This raw difference therefore has little meaning and these two factors must be controlled for.

To control for the reading frame effect, hits to the stop codon containing motifs were predicted separately in each reading frame and the number of predicted hits scaled by the number of motifs that can function in that frame. This scaling is not uniform (i.e. TGAAGA, TGAAGC, TGAAGG, TGAGAA cannot function in the +0 frame, AATGAC, AGTGAC, GATGAA in the +1 frame or CTGAAG, GTGAAG in the +2 frame; frames denote motif start position relative to the ORF). In the +0 frame, only 5/9 motifs contribute to hits and we are therefore in effect only sampling 5/9 of potential hits that a motif not containing a stop codon could contribute. The raw hit count was therefore multiplied by 9/5 (and similarly 9/6, 9/7 for the +1, +2 frames).

Second, to control for the number of motifs per class (stop codon containing motifs or motifs not containing stop codons), the frame-normalised total hits in each class were divided by the number motifs in each class (N = 9 for stop codon containing and N = 75 for motifs that contain no stop codons). This provided the total hits per frame per motif for both classes of motif. This total was further normalised to give the number of hits per 1,000 bp of protein-coding exonic sequence to give a normalised per motif per 1,000 bp hits (NMH) for all coding exons. An NMH = 2 for stop codon containing motifs, for example, means that on average there exist two hits to each stop codon containing motif per 1,000 bp of lincRNA sequence.

We find the stop codon containing INT3 ESE NMH = 0.928 is significantly greater than that for 1,000 sets of dinucleotide-matched and stop-codon matched (that is, the same number of motifs contain a stop codon for each iteration) pseudo-ESE motifs (median simulant NMH = 0.647, $P \approx 0.009$, one-tailed empirical P-value). Whilst motifs that do not contain stop codons are also found significantly more frequently than dinucleotide matched pseudo-motifs (NMH = 0.596, median simulant NMH = 0.465, $P \approx 9.99 \times 10^{-4}$, one-tailed empirical P-value), the difference between the two NMH values for the real INT3 ESE motifs argues that, if anything, stop codon containing motifs are more frequent and not avoided.

Despite this greater per motif use of the stop codon containing motifs, this could be explained if the stop codon containing motifs better match the nucleotide composition of protein-coding exons. Is the difference therefore greater than expected? We calculated the ratio between NMH value of stop codon containing motifs to that of motifs that contain no stop codons ($0.928/0.596 \approx 1.559$) and asked whether this is greater than expected by chance. To define chance, we considered the 1,000 sets of simulated pseudo-ESEs and calculated the same ratio. The ratio for real ESEs is not significantly greater than the equivalent ratio for the null pseudo-ESE motif sets (median simulant NMH ratio = 1.399, $P \approx 0.257$, one-tailed empirical P-value).

Thus, although stop codon containing ESEs are per motif more frequent, this result suggests the increased usage between the two classes if not significantly greater than expected. Repeating the analysis for a total of 10 runs to control for paralogous family member choice, per motif stop codon containing ESE enrichment remains significantly greater than controls (median $P \approx 0.009$, one-tailed empirical P-value) but not significantly greater than per motif usage of the remaining ESEs (median $P \approx 0.257$, one-tailed empirical P-value, Supplementary Table 2) and results are therefore not biased to sequences interrogated.

If stop codon containing ESEs were of lesser quality and under weaker selection or selected against, there should be a depletion of the stop codon containing motifs relative to motifs not containing stop codons. We find no evidence this is the case and no conclusive evidence to argue the depletion of stop codons is a result of avoiding employing stop motifs as a consequence of being less functional.

Supplementary Text 3: The density of high quality, low false-positive stop codon containing ESE motifs increases as flanking intron size increases

Results suggest the relative usage of stop codon containing motifs per motif is not significantly greater than for motifs of similar dinucleotide content. However, the per motif frequency may not be the most informative measure if the quantity of splice information incorporated is important for ensuring accurate splicing. For example, by having more contributing motifs, the combined quantity of splice information encoded by ESEs not containing stop codons may be important. Thus, total ESE frequency (or density) rather than per motif frequency could be more informative.

ESE density is known to be positively correlated with intron size in protein-coding genes (Dewey et al. 2006; Caceres and Hurst 2013; Wu and Hurst 2015), thought to be a result of reinforcement selection increasing the quantity of splice information to distract SR proteins away from possible cryptic splice sites (Wu and Hurst 2015). Any differences how stop codon containing motifs and the remaining motifs are employed as the flanking intron size increases may therefore provide further insight.

We first check whether ESE density in general increases as flanking intron size increases in our protein-coding exon dataset after grouping paralogous family members (Dewey et al. 2006; Caceres and Hurst 2013; Wu and Hurst 2015). We find this to be the case (ρ = 0.198, P = 1.04×10^{-24}, Spearman's rank correlation, Supplementary Texts Figure 1A). As ESEs typically reside in exon flanks in protein-coding genes and therefore more likely to be splice related, we limited sequences to only the 5' and 3' flanks (nucleotides 2-69 from exon boundary) of sequences greater than 207 nucleotides in length (to restrict sequences to those with both 5'/3' flanks and core regions). By doing this, we also control for the quantity of sequence in which the ESEs can reside, and thus ESE density directly reflects how many ESEs are incorporated. Again, in the exon flanks, we find significant positive correlations with intron length (ρ = 0.136, P = 2.79×10^{-12}, Spearman's rank correlation). Results are not subject to biases as a result of picking one member at random from each paralogous family grouping as we find a similar significant positive correlation when using all sequences (ρ = 0.158, P = 2.05 $\times 10^{-61}$, Spearman's rank correlation).

If stop codon containing ESEs are less functional and avoided, it would be expected that they are used less frequently as flanking intron size increases. We asked whether the usage of stop codon containing ESEs and ESEs containing no stop codons differs as intron size increases. ESEs were grouped into stop codon containing motifs and others and densities calculated for the two classes. To control for the number of motifs in each class and the reading frame restrictions on the stop codon containing ESEs, we only compare the correlations between densities and intron size for each the two ESE motif classes rather than directly comparing densities themselves. We find correlations are significantly positive for both stop codon

containing (ρ = 0.139, P = 1.07 $\times$ 10^{-12}, Spearman's rank correlation) and the remaining ESEs (ρ = 0.190, P = 2.17 $\times$ 10^{-23}, Spearman's rank correlation) (Supplementary Texts Figure 1B), suggesting the usage of both classes of ESE increases as greater quantities of splice information are required.

Is the density of stop codon containing motifs significantly positively correlated when restricted only to exon flanks? Again, correlations of both stop codon containing (ρ = 0.078, P = 4.586 $\times$ 10^{-5}, Spearman's rank correlation) and remaining ESEs (ρ = 0.132, P = 1.04 $\times$ 10^{-11}, Spearman's rank correlation) with intron length are significantly positive. Interestingly, the density of stop codon containing motifs does increase significantly slower than for the remaining ESEs (Z = 5.176, P = 2.26 $\times$ 10^{-7}, two-tailed Z-tests of equivalency in exon flanks) meaning it could be the case that the ESEs not containing stop codons are more specialist (they have, for example, greater binding affinities), or stop codon containing ESEs have additional constraints (which could be the case due to reading frame requirements). Importantly, evidence suggests stop codon containing ESEs are not avoided as splicing becomes more difficult.

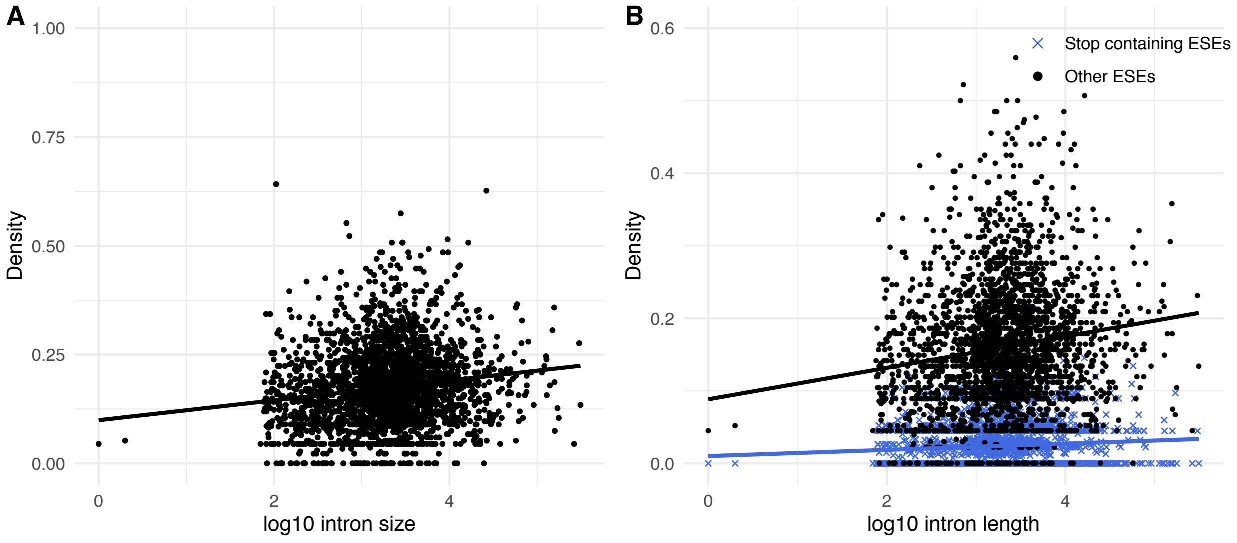

Supplementary Texts Figure 1: The log10 median lengths for introns versus the density of INT3 ESE motifs for the flanking 2-69 nucleotides of (A) all ESEs combined and (B) ESEs grouped as stop codon containing and the remaining ESEs. In all cases, correlations between flanking intron size and densities are significantly positively correlated (P < 0.01, Spearman's rank correlations).

Supplementary Text 4: Stop codon containing ESE motifs can overlap with fewer motifs than the remaining ESEs, but not less than dinucleotide matched controls

The result in Supplementary Text 3 suggests that while stop codon containing motifs are not avoided as splicing becomes more difficult, motifs not containing stop codons are increasingly used over those containing stop codons. Are there constraints at the sequence level such that ESEs containing no stop codons are used more frequently? If it is the case, stop codon containing motifs may still increase within increasing flanking intron size because they provide splice function, but usage may be restricted. In such a case, it could again help to explain the stop codon depletion beyond protein-coding constraints predicted under our transfer selection model.

We note that we observe an increase in ESE density for both stop codon containing motifs and others even when for exon flank sequences the size of the sequence scanned is controlled. In this scenario, for ESE density to increase there is an increased probability that motifs will have to overlap to include the splice information. We calculated the number of overlapping motifs and non-overlapping motifs in each exon (both classes of motifs combined), overlapping defined as at least one nucleotide shared between two or more motifs. We find a weak yet significant correlation between both the raw number of overlapping motifs (ρ = 0.065, P = 8.127 $\times$ 10^{-4}, Spearman's rank correlation) and the proportion of overlapping motifs within a sequence (ρ = 0.077, P = 1.069 $\times$ 10^{-4}, Spearman's rank correlation) as flanking intron size increases, suggesting more overlapping does occur as more splice information is required.

However, to overlap in protein-coding sequence the motifs in addition to functioning as RBP-binding sites must, at a minimum, also encode the correct amino acids (although other constraints e.g. RNA secondary structures are likely to be imposed). In protein-coding sequence, this is more difficult if a motif contains a stop codon. For example, suppose there exists the sequence NNA|AGA|TTA|AGA. A **T** $\rightarrow$ **G** mutation creates two ESE motifs GATGAA and TGAAGA but also creates a premature stop codon. However, a **T** $\rightarrow$ **C** mutation does not alter amino acid content nor generate an in-frame stop codon, whilst also creating ESE motifs AAGATC and TCAAGA that could serve as potential functional SR protein binding sites. If there exists a selection pressure to maximise splice information, the only useful motifs would be those containing no stop codon (the probability the mutation reaches fixation would then be determined by whether the new binding motifs provide sufficient functional benefit). At the sequence level, stop codon containing ESEs may therefore be less preferable at higher ESE densities not because they are poorer functioning motifs (e.g. reduced ability to ensure accurate splicing), but because they are more difficult to include.

To establish whether this can explain the depletion of stop codons in ESEs, we must first ask whether stop codon containing motifs themselves are inherently more difficult to overlap –

that is, are stop codon containing motifs less likely to be used because their dinucleotide content prevents them from overlapping with other motifs as frequently.

For each INT3 motif, we calculated the number of other motifs (including the focal motif) that overlap the focal motif by at least two nucleotides. For example, the two motifs AT**GTAA** and **GTAA**TA share a four-nucleotide overlap. The mean number of motifs a stop codon containing motif can overlap with (28.667) is fewer than for the motifs containing no stop codons (36.800). Is this attributable to the dinucleotide content of the motifs? Calculating the mean number of overlaps for the 1,000 sets of dinucleotide- and number of stop codon-matched null motif sets (stop codon-matched such that an equal number of pseudo ESE motifs per iteration contain stop codons), the real stop codon containing motifs do not overlap with significantly fewer motifs when compared with the controls ($P \approx 0.732$, one-tailed empirical P-value). A fold-enrichment overlap score (FEO) of 0.083 (calculated as per FE using the real stop codon containing mean overlap and simulant ESE mean overlap) confirms the insignificant difference.

Motifs containing no stop codons, however, can overlap with a significantly greater number of motifs than expected (FEO = 0.333, $P \approx 9.99 \times 10^{-4}$, one-tailed empirical P-value). Moreover, this difference between the number of overlaps between the stop codon containing and remaining ESEs is also greater than expected ($P \approx 0.031$, one-tailed empirical P-value). Using the RESCUE set of ESEs, the number of motifs a motif can overlap with is significantly greater than expected for both stop codon containing (75.833, $P \approx 0.003$, one-tailed empirical P-value) and the remaining motifs (77.390, $P \approx 9.99 \times 10^{-4}$, one-tailed empirical P-value), with this difference not significantly greater than the control motifs ($P \approx 0.148$, one-tailed empirical P-value), suggesting the ability to overlap is not subject to motif set bias.

Taken together, rather than the dinucleotide content of stop codon containing motifs making it more difficult to overlap, motifs containing no stop codons can consistently overlap more frequently with other motifs. Thus, the relative increase in usage of ESEs containing no stop codons with intron size is unlikely to be a result of difficulties including a stop codon containing motif where two are required to overlap.

Supplementary Text 5: A greater frequency of ESE motifs containing stop codons when required to overlap another ESE motif from that expected argues against a stop codon depletion in the ESEs due to an inability to be combined with other motifs

The result in Supplementary Text 4 raises an interesting question. Could the composition of real ESE motif set be due to an enrichment of motifs containing no stop codons that are more readily able to overlap and as a consequence depleted for stop codon containing motifs, independent of any protein-coding constraints? If so, we expect stop codon containing motifs to be used less frequently when overlapping another motif in exonic sequences.

We therefore predicted hits to all motifs within protein-coding exons and for each motif asked whether it overlapped another. For both overlapping motifs and non-overlapping motifs, we then asked what proportion contained stop codons. In this way, we can establish whether stop codon containing motifs are used less frequently when involved in an overlap, controlling for the fact these motifs are fewer in number. For the INT3 set, we find the opposite to be true – a slightly higher proportion of motifs contain a stop codon if overlapping another motif (0.106) than if not overlapping (0.098). Although small, this is a highly significant increase (χ^2 = 49.863, P = 1.649×10^{-12}, chi-square test of raw frequencies using non-overlap frequency as the expected frequency). A similar significant increase is observed for the RESCUE set (overlap stop proportion: 0.143, non-overlap stop proportion: 0.119, χ^2 = 1041.418, P = 1.649×10^{-12}, chi-square test).

Thus, although stop codon containing motifs are intrinsically more difficult to overlap (see Supplementary Text 4), we instead find a significant increase in usage from that expected if a stop codon containing motif overlaps another motif. This argues against the hypothesis that stop codon containing motifs are harder to include in overlapping motifs in the real sequences. The depletion of stop codons in ESEs is unlikely to result from stop codon containing motifs being of less utility in forming part of a larger binding motif. This result would also suggest that much of the increase in ESE density as intron size increases is likely due to singular motifs. Together, these results argue against stop codon containing ESEs being of poorer function.

Supplementary Text 6: The depletion of stop codons in lincRNA sequences is not a result of biases due to the sequences chosen paralogous family groupings

To dismiss the possibility that the depletion of stop codons in lincRNA is due to biases as a result of the random paralogous family member chosen, we ran the analyses shuffling the Cabili et al. (2011) lincRNA sequences 10 times in total. The depletion of stop codons remains significant in all cases (median $P \approx 9.99 \times 10^{-4}$; median real SCD = 0.130, median of median simulated SCDs = 0.155, median FE = -0.162, Supplementary Table 3). The same control applied to the pairwise test in the main text also holds (median all depletions $P \approx 0$, median significant depletions $P \approx 7.09 \times 10^{-201}$, Supplementary Table 4). The depletion also exists if all sequences are considered and not grouped by family ($P \approx 9.99 \times 10^{-4}$, one-tailed empirical P-value, N = 4,646, SCD = 0.135, median simulated SCD = 0.158, FE = -0.149, Supplementary Table 5). We can therefore eliminate biases due to the individual sequences used as the reason for the depletion of stop codons seen in lincRNAs.

Supplementary Text 7: The depletion of stop codons in lincRNA sequences is robust in the second set of independently derived lincRNA sequences

To verify the depletion of stop codons in lincRNA sequences by eliminating biases due to the total set of lincRNA sequences chosen, we repeated the analysis using a more recently derived set of GENCODE sequences reannotated by RNA Capture Long Seq (CLS) (Lagarde et al. 2017). CLS enables manual-quality full-length annotations at high throughput levels and enables a quality assessment of protein-coding potential (Lagarde et al. 2017). A minority of these sequences had protein-coding potential, but none had peptide-based evidence of translation. This set therefore provides a second "clean" dataset with minimal protein-coding contamination.

Results are qualitatively similar to those when using the Cabili et al. (2011) sequences. In the dataset as a whole, stop codons are depleted when compared with shuffled versions (FE = -0.169, $P \approx 9.99 \times 10^{-4}$, one-tailed empirical P-value, Supplementary Table 6). Similarly, 91.23% (416/456) of the sequences have a stop codon depletion when compared with randomisations for the same gene, again a significant excess above null ($P \approx 0$, one-tailed exact binomial test, null probability of success = 0.5, Supplementary Table 7). Of these, 206/456 (45.18%) have a significant depletion ($P = 2.33 \times 10^{-139}$, one-tailed exact binomial test, null probability of success = 0.05). These results, as per the Cabili et al. (2011) set of sequences, are not biased by sequence chosen (Supplementary Tables 6-7). Further, the SCD in exons is less than in introns of the same gene for 325/456 (71.27%) genes ($P = 2.33 \times 10^{-139}$, one-tailed exact binomial test, null probability of success = 0.5).

Supplementary Text 8: The stop codon depletion in lincRNAs is not owing to hidden ORF contamination

Could the apparent lack of stop codons in lincRNAs be biased by a subset of sequences being under strong selection to avoid stop codons, as these sequences contain true but unrecognised ORFs?

The initial quality control of the lincRNA datasets argues against this. However, we also consider only the sequence upstream of the first annotated ATG (in any frame) in lincRNAs which should be devoid of protein-coding potential (although we cannot eliminate cases where transcription start sites are 5’ to the annotated sequence). Again, we find a depletion of stop codons compared with the randomly shuffled nulls of these upstream sequences, robust to differing lengths of sequence before the ATG (median $P \approx 9.99 \times 10^{-4}$, one-tailed empirical P-value, median real densities = 0.089, median simulant densities = 0.114, Supplementary Table 8). The stop codon depletion in lincRNA is therefore not parsimoniously explained as simple annotation artefact.

Supplementary Text 9: The stop codon density in multi-exon 5’ untranslated sequences is significantly greater than in single-exon sequences, but not after controlling for nucleotide composition

Under our model, it could be expected that the 5’ untranslated regions (UTRs) should behave similarly to lincRNA sequences as these should have no underlying coding potential. Also, the first intron is often close to the ATG and hence to the UTR. Therefore, we hypothesise that those UTR sequences for multi-exon sequences should have significantly lower SCD than those for single-exon sequences. Considering only 5’UTRs of length greater than 50 nucleotides and after picking one sequence per paralogous family, this is what we find ($P = 2.218 \times 10^{-5}$, Wilcoxon rank sum test, median single-exon 5’ UTR SCD = 0.074, median multi-exon 5’ UTR SCD = 0.066).

However, when comparing the FE scores after generating null sequences by randomly shuffling the nucleotides of the UTR sequences, we find no significant difference between FE scores of the real lincRNA and the simulants (P = 0.203, Wilcoxon rank sum test). Thus, it appears that the 5’ UTR sequences of multi-exon sequences are less conducive to incorporating stop codons than expected. Surprisingly, if anything it is the UTRs of single-exon sequences that are more deviated from the null (median single-exon 5’ UTR FE = -0.150, median multi-exon 5’ UTR FE = -0.141).

At first sight, this appears to contradict our hypothesis. However, upon closer inspection, it is the single-exon UTRs with significantly higher ESE density ($P = 3.667 \times 10^{-9}$, Wilcoxon rank sum test, median single-exon 5’ ESE density = 0.113, median multi-exon 5’ ESE density = 0.092). Consistent with our model, it is therefore the sequences with greater ESE density that have greater negative deviations from expected in SCD. Why the 5’ UTR sequences of single-exon sequences contain more ESEs than those of spliced sequences is unanswered but could be due to the additional functional roles of ESEs beyond splicing (Savisaar and Hurst 2016).

Supplementary Text 10: Enrichment of stop codons in non-ESE lincRNA sequence may prevent inappropriate SR-protein binding

Is the enrichment of stop codons in lincRNA sequence that is not predicted to be ESE genuine? If not, and a consequence of the remaining sequence being conducive to generating stop codons, the SCD of the remaining sequence should be similar to a sequence in which motifs with similar dinucleotide content have been removed. However, if it is, the remaining sequence should have a greater SCD after removal of dinucleotide-matched controls. In this test, the dinucleotide-matched control motifs must also be matched in stop codon frequency otherwise the simulated remaining sequence may retain greater/fewer stop codons as there are more/fewer motifs with stop codons to potentially remove. After removal of motifs from the combined ESE set, the remaining sequence has a higher SCD than after removing sequence matching the dinucleotide- and stop codon-number matched control motifs (FE = 0.116, $P \approx 0.003$, one-tailed empirical P-value). This result is not affected by the randomly paralogous family member sequences chosen (Supplementary Table 9). This result is therefore consistent with a genuine increase in stop codons in lincRNA outside of ESE motifs.

Why then might the remaining sequence be enriched for stop codons? One explanation could be that there exists a selective pressure to include more stop codons in non-ESE sequence at exon cores or 3' flanks such that incorrect SR protein binding is less likely as the remaining sequence "appears" less like ESE. If such a selection pressure exists, the difference in SCD between ESE hits and the remaining sequence should be greater than similar comparisons when predicting hits to control motifs. This is what we find. The SCD in ESE hits is 0.087 and the remaining sequence 0.113. The ratio of SCD of ESEs to non-ESEs is $0.087/0.113 \approx 0.770$, significantly smaller than the equivalent ratio for the control motifs (median control motif proportion = 0.908, $P \approx 9.99 \times 10^{-4}$, one-tailed empirical P-value), indicating that there is indeed a larger difference in SCD in real sequence than expected. Whilst indicative, this result is also compatible with selection to incorporate stop codons that become premature termination codons (PTCs), thereby making the transcript subject to nonsense-mediated decay (NMD) if erroneously recruited to the translation machinery (Niazi and Valadkhan 2012). We make no further inferences as to reasons for the increased SCD, but simply suggest there may be several regulatory mechanisms that would benefit from such selection.

Supplementary Text 11: The stop codon depletion is not a result of lincRNA sequences avoiding the use of stop codon containing ESEs

Our results suggest that despite the lack of translational constraint, stop codons are found less frequently than expected in lincRNA. While this is consistent with our transfer selection model, it could also be explained if, for some unknown reason and unlike protein-coding sequences, stop codon containing ESEs are less functional or avoided in lincRNAs. If this is the case, then stop codon containing motifs should be found less frequently per motif than the other ESE motifs and should be under-employed compared with sets of nucleotide composition-matched controls.

To address this, we calculated the raw number of hits within the lincRNA sequences to both stop codon containing ESE motifs and the remaining ESE motifs from the INT3 data set as per protein-coding sequences in Supplementary Text 2. From this, we calculated the normalised number of hits per motif per 1,000 bp (NMH) in lincRNA exons. With no reading frame constraints in lincRNA, the first part of the normalisation divided the total raw hits in each class by the total number of motifs contributing to each class (N = 9 stop codon containing ESE motifs, N = 75 other ESE motifs), to give the number of hits per ESE in each class. This value was further normalised to give the number of hits per 1,000 bp of exonic sequence.

We find that the stop codon containing INT3 ESEs have more hits within lincRNAs (NMH = 0.464) than for dinucleotide-matched simulant sets of pseudo-ESE motifs (see Supplementary Table 10), although not significantly so (median simulant NMH = 0.409 hits per stop codon containing motif, $P \approx 0.113$, one-tailed empirical P-value). We therefore conclude that the stop codon containing ESE motifs are not avoided when compared to null expectations. The NMH for the real INT3 motifs not containing a stop codon is also greater than for the pseudo-ESE motifs that do not contain stop codons (NMH = 0.489), but again not significantly so (median simulant NMH = 0.450 hits per motif for motifs not containing stop codons, $P \approx 0.055$, one-tailed empirical P-value).

While these results indicate no avoidance of stop codon containing ESE motifs in lincRNA, they are together surprising as we expect ESE motifs to be enriched in lincRNA compared to nucleotide-matched controls. When both the stop codon containing motifs and those containing no stop codons are combined, we do find the real ESE motifs are found more frequently than the simulant motifs in lincRNA ($P \approx 0.039$, one-tailed empirical P-value).

While neither of the sub-groups (those hexamers with a stop codon and those without) is significantly enriched in isolation, the data suggests a possible greater enrichment of the motifs that do not contain stop codons (these motifs are borderline significantly enriched). Might this indicate possible preferential usage of the motifs that do not contain a stop codon? To address we take the ratio between the hits per motif seen in the two groups (NMH for stop

codon containing motifs = 0.464, for motifs without a stop codon = 0.489, ratio = 0.949) and ask whether this is lower than expected by chance.

To define chance, we consider sets of simulated ESEs in which the dinucleotide content of the real set is maintained but ensuring the total number of stop codons within the set of simulated ESEs equals that in the real ESEs. For each simulant set of ESEs we again split the motifs into two groups, those with stop codons and those without. For each class of pseudo-ESE, we determine the hits per motif in lincRNA and the ratio of average hits per motif within each group (those with a stop codon to those without). By repeating multiple times, we can then define the null distribution of values of the above ratio controlling for dinucleotide content of ESEs. From this, we determine that the observed ratio is not significantly lower than expected by chance, even employing a one-tailed test ($P \approx 0.656$ one-tailed empirical P-value, median simulant ratio = 0.905).

We conclude that there is no evidence stop codon containing ESEs are underemployed, given both the commonality of such ESEs and the dinucleotide content of ESEs. Results are not subject to biases due to the random sequences chosen (Supplementary Table 10). Furthermore, this is also not a result of the ESEs used. Using the RESCUE ESE dataset containing more motifs (Supplementary Table 11), we find both hits to stop codon containing motifs (NMH = 0.444, median simulant NMH = 0.388, $P \approx 0.004$, one-tailed empirical P-value) and hits to those motifs not containing stop codons (NMH = 0.413, median simulant NMH = 0.376, $P \approx 0.003$, one-tailed empirical P-value) are significantly greater than matched pseudo motifs, while the ratio between the NMH values is not significantly greater than expected (ratio = 1.076, median simulant ratio = 1.030, $P \approx 0.747$, one-tailed empirical P-value). This suggests that both the ESE motifs containing stop codons and those not containing stop codons are enriched in lincRNAs and that neither class is significantly more or less enriched than the other. The depletion of stop codons in lincRNA is unlikely to be a result of underuse of specific ESEs or avoidance of stop codon containing ESEs in lincRNA.

Supplementary Text 12: Stop codon containing ESEs are not avoided in lincRNA as intron size increases

Are stop codon containing ESEs functional in lincRNA sequences? As with protein-coding sequences (see Supplementary Text 3), one indication of their functionality would be an increased density as flanking intron size increases. Correlations between ESE density and intron size have previously been documented by Schuler et al. (2014) using the RESCUE set of motifs (Fairbrother et al. 2004). We find a similar trend for both full sequences (ρ = 0.190, P = 4.37×10^{-17}, Spearman's rank correlation) and when restricted to exon flanks (ρ = 0.010, P = 7.26×10^{-5}, Spearman's rank correlation) for our dataset (N = 1,919 lincRNA sequences).

Does the use of stop codon containing ESEs increase with intron size? As with protein-coding sequences, we again find a significant positive correlation in full sequences (ρ = 0.126, P = 4.37×10^{-17}, Spearman's rank correlation) and flanking regions (ρ = 0.049, P = 0.049, Spearman's rank correlation, Supplementary Texts Figure 2), arguing that the motifs are indeed employed and functional. Although the signal is much weaker when restricted to flanks, only the density of motifs rather than exon location appears important in lincRNA (Schuler et al. 2014). Thus, with both the flanking regions and full sequences both displaying significantly positive trends, these results argue that stop motifs are likely functional. As per protein-coding genes, the density of ESEs containing no stop codons is also significantly positively correlated within flanking intron size (all sequence: ρ = 0.180, P = 1.94×10^{-15}; flanks: ρ = 0.087, P = 4.28×10^{-4} Spearman's rank correlations).

With evidence arguing the enrichment of ESEs near exon ends is indicative of functionality in CDS (Fairbrother et al. 2004; Carlini and Genut 2006; Parmley et al. 2006; Parmley et al. 2007; Ke et al. 2011; Sterne-Weiler et al. 2011; Caceres and Hurst 2013; Ramalho et al. 2013; Savisaar and Hurst 2018) and lincRNAs (Schuler et al. 2014; Haerty and Ponting 2015), this is consistent with stop codon containing ESEs likely being functional and therefore the interpretation of low SCD is the need to preserve ESEs, in which stop codons are depleted.

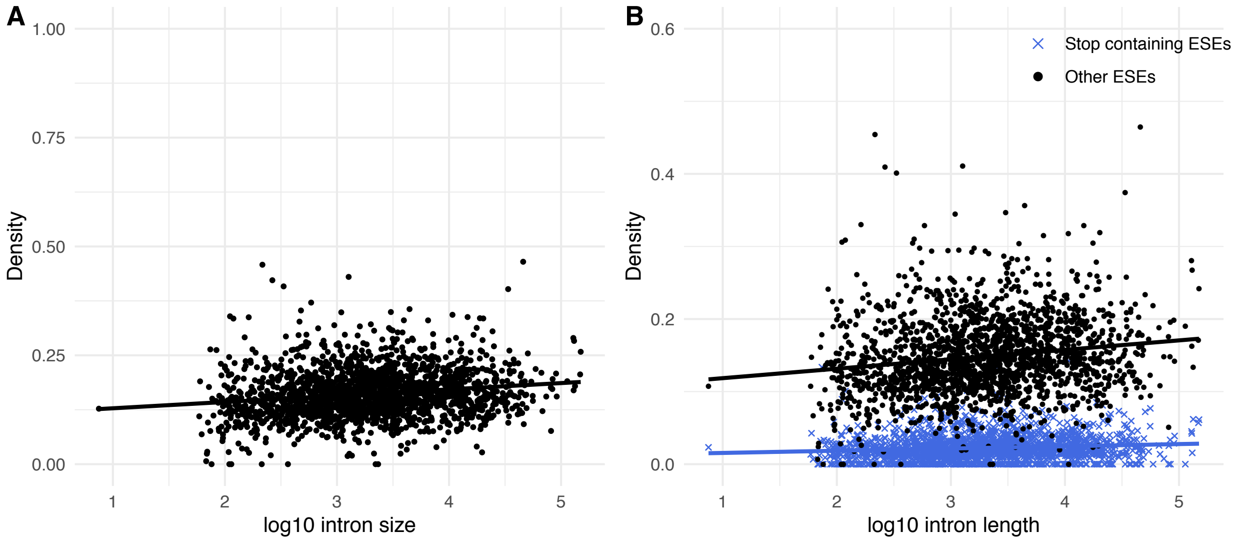

Supplementary Texts Figure 2: The log10 median lengths for introns versus the density of INT3 ESE motifs for the whole exon when (A) all ESEs are combined and (B) ESEs are grouped as stop codon containing and those containing no stop codons. In all cases, correlations between flanking intron size and densities are significantly positively correlated (Spearman's rank correlations). Correlations of densities in whole exons rather than exon flanks are shown as ESE location is not considered as such an important predictor of evolutionary rate in lincRNA (Schuler et al. 2014).

Supplementary Text 13: The high purine content of ESEs is consistent with a model in which ESEs are highly non-intronic, making the depletion of purine-rich stop codons particularly noteworthy

That both ESEs (Xu et al. 1993; Dirksen et al. 1994; Tanaka et al. 1994; Gersappe and Pintel 1999; Fairbrother et al. 2002; Caceres and Hurst 2013) and stop codons are purine-rich makes the depletion of stop codons in ESEs more noteworthy. With skewed nucleotide usage at exon ends in both protein-coding and lincRNA sequences, is the high purine content of ESEs in itself a defining feature of ESEs? The presence of uracil residues within polypurine sequences has been shown to reduce splicing ability (Tanaka et al. 1994). Thus, purine-richness may simply be a requirement of SR protein binding - the binding sites of the SR protein SF2/ASF, for example, are 80% purine (Graveley 2000).

As vertebrate genes are characterised by short exons dispersed between longer introns (Zhang 1998; Sakharkar et al. 2005), the more distinguished a motif is within its surrounding pre-mRNA transcript sequence, the less erroneous binding to intronic sequence should occur. An alternative, but not necessarily mutually exclusive model, is therefore one in which the purine content helps to distinguish ESEs. This model makes several basic predictions. First, exonic sequences should differ in purine content to introns. In other words, if purine content helps to define ESEs as exonic, in the first instance one would expect that exons themselves have increased purine content when compared with intronic sequence. Second, random intronic motifs should have reduced purine content compared with the real motifs, minimising the chance of inappropriate SR protein binding to introns.

We therefore calculated the purine content for coding exons and their corresponding introns, with genes considered as part of the same paralogous family as a single data point (N = 5,620). As expected, the purine content for exons (median purine content = 0.523) is significantly greater than that of introns (median purine content = 0.490) ($P < 2.2 \times 10^{-16}$, paired Wilcoxon signed-rank test). In general, exons are more purine-rich than introns (Supplementary Texts Figure 3). This differentiation suggests that by being purine-rich, ESEs can be seen as non-intronic sequence. However, this increased exonic purine content may simply be a consequence of the ESEs situated within the exons. After removing all possible motifs using the combined ESE set exonic purine content remains significantly greater ($P < 2.2 \times 10^{-16}$, paired Wilcoxon signed-rank test, median exon purine content = 0.456, median intron purine content = 0.438), suggesting even without ESEs exons are different in terms of purine content.

Are then, ESEs also differentiated further from exons because of their purine content? Using the terminal 50 nucleotides of exon sequences, Caceres and Hurst (2013) find ESEs to have significantly higher purine content. This result holds in our dataset when employing exons longer than 100 nucleotides (N = 5,032 data points) ($P < 2.2 \times 10^{-16}$, paired Wilcoxon signed-rank test). However, this differentiation should not be limited simply to exon ends - a

difference in purine content should extend throughout the whole exon as ESEs need to differentiate from surrounding exonic sequence. Using the INT3 set of ESEs, we find that the purine content of sequence that overlaps an ESE is significantly greater than that of non-ESE exonic sequence ($P < 2.2 \times 10^{-16}$, paired Wilcoxon signed-rank test). Thus, whilst the purine content of exons tends to be greater than that of introns, ESEs are further differentiated from the surrounding sequence. This result is therefore consistent with the ESE purine content defining ESEs as not only non-intronic, but highly non-intronic and may therefore act as a marker to differentiate key binding sites.

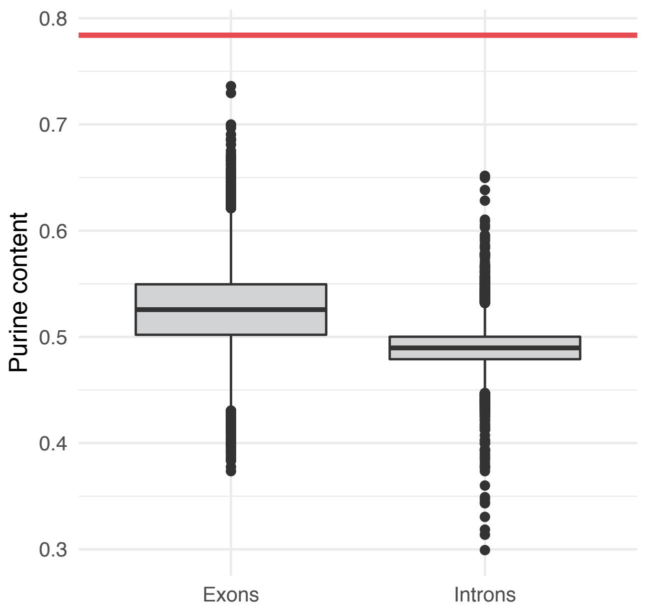

Supplementary Texts Figure 3: The purine content of both exon and introns, with the purine content of INT3 ESEs shown by the horizontal line. Exons in general tend to have higher purine than introns. The purine content of ESEs differentiates them from both surrounding exonic and intronic sequence.

This purine-richness should therefore make ESEs all the rarer within the intronic sequence, possibly such that ESEs are not frequently found within introns to prevent inappropriate binding. This argument is logical – ESEs, as motifs functioning within exons, should be less abundant in intronic sequence. However, it has been documented that ESEs may have functional roles in introns; increases in ESE density have been documented in introns with weak donor sites, suggesting ESEs that help to splice weak donor sites may exist in introns rather than exons (Wu et al. 2005), whilst ESEs in introns have also been shown to have repressor abilities (Kanopka et al. 1996; McNally and McNally 1998). Does then, the purine content of ESEs differentiate them from random intronic motifs for the probability of an SR protein inappropriately binding within an intron to be reduced?

We generated 1,000 sets of random hexamers from intronic sequence, picking one gene at random from those considered as part of a paralogous family. We then asked whether the

purine content of these random motifs differed significantly from the real ESE motifs. We find this is to be the case ($P \approx 9.99 \times 10^{-4}$, one-tailed empirical P-value) – no set of hexamers has purine content close to that of ESEs (Supplementary Texts Figure 4A). Thus, hexamers with the nucleotide content found within ESEs are highly unlikely to be frequently found within introns, making them ideal candidates to ensure the SR proteins correctly locate and bind exclusively exonic sequence in proximity to splice sites.

Given the above result, it is interesting to ask whether there is a particular bias of A/G nucleotides within ESEs that discriminates them from intronic hexamers. We therefore calculated the proportions of each nucleotide in ESEs and the random hexamers derived from intronic sequence. We find a striking difference in both A and G content when compared with these motif sets (Supplementary Texts Figure 4B), finding no sets of random intronic hexamers with either A or G content greater than that of the real ESEs ($P \approx 9.99 \times 10^{-4}$, one-tailed empirical P-values). Thus, it is the combination of both A and G nucleotides found less frequently within introns that differentiates ESEs from intronic sequence.

By having high purine content, ESEs therefore differ significantly from both intronic and exonic sequence, making it less likely SR proteins will bind off-target motifs. Purine content may then act to differentiate such motifs. Despite this, ESEs may have functional repressive roles if present in introns (Kanopka et al. 1996) and may complicate this issue. However, this model still predicts that the purine content would help differentiate such motifs from surrounding intronic sequence and therefore still preventing inappropriate binding. We make no further attempts to address this issue, but simply conclude that the purine content is consistent with helping ESEs look different from the surrounding sequence.

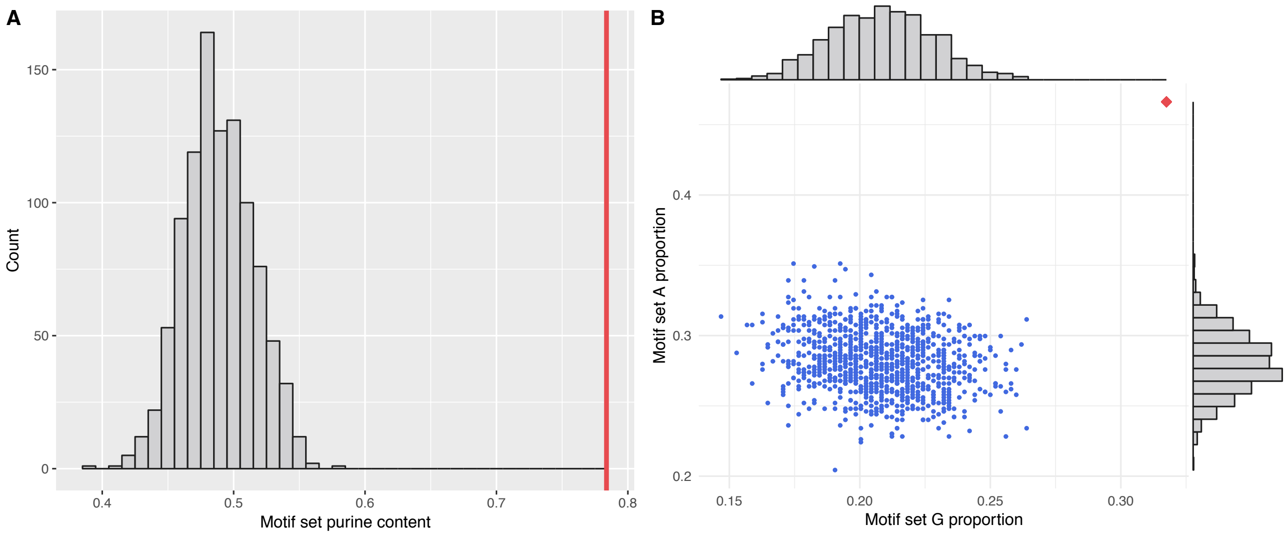

Supplementary Texts Figure 4: (A) The purine content of INT3 ESEs (vertical red line) is significantly greater than the purine content of random sets of intronic hexamers of equal number. (B) Both the A and G content of ESEs is greater than that of the random intronic hexamer sets. We find no set with both A and G content higher than that of the real INT3 ESE set.

References

Cabili MN, Trapnell C, Goff L, Koziol M, Tazon-Vega B, Regev A, Rinn JL. 2011. Integrative annotation of human large intergenic noncoding RNAs reveals global properties and specific subclasses. *Genes Dev.* 25:1915-1927.

Caceres EF, Hurst LD. 2013. The evolution, impact and properties of exonic splice enhancers. *Genome Biol* 14:R143.

Carlini DB, Genut JE. 2006. Synonymous SNPs provide evidence for selective constraint on human exonic splicing enhancers. *J. Mol. Evol.* 62:89-98.

Dewey CN, Rogozin IB, Koonin EV. 2006. Compensatory relationship between splice sites and exonic splicing signals depending on the length of vertebrate introns. *BMC Genomics* 7:311.

Dirksen WP, Hampson RK, Sun Q, Rottman FM. 1994. A purine-rich exon sequence enhances alternative splicing of bovine growth hormone pre-mRNA. *J. Biol. Chem.* 269:6431-6436.

Fairbrother WG, Yeh RF, Sharp PA, Burge CB. 2002. Predictive identification of exonic splicing enhancers in human genes. *Science* 297:1007-1013.

Fairbrother WG, Yeo GW, Yeh R, Goldstein P, Mawson M, Sharp PA, Burge CB. 2004. RESCUE-ESE identifies candidate exonic splicing enhancers in vertebrate exons. *Nucleic Acids Res.* 32:W187-190.

Gersappe A, Pintel DJ. 1999. CA- and purine-rich elements form a novel bipartite exon enhancer which governs inclusion of the minute virus of mice NS2-specific exon in both singly and doubly spliced mRNAs. *Mol. Cell. Biol.* 19:364-375.

Graveley BR. 2000. Sorting out the complexity of SR protein functions. *RNA* 6:1197-1211.

Haerty W, Ponting CP. 2015. Unexpected selection to retain high GC content and splicing enhancers within exons of multiexonic lncRNA loci. *RNA* 21:333-346.

Hoffman BE, Lis JT. 2000. Pre-mRNA splicing by the essential Drosophila protein B52: tissue and target specificity. *Mol. Cell. Biol.* 20:181-186.

Kanopka A, Muhlemann O, Akusjarvi G. 1996. Inhibition by SR proteins of splicing of a regulated adenovirus pre-mRNA. *Nature* 381:535-538.

Kawano T, Fujita M, Sakamoto H. 2000. Unique and redundant functions of SR proteins, a conserved family of splicing factors, in Caenorhabditis elegans development. *Mech Dev* 95:67-76.

Ke S, Shang S, Kalachikov SM, Morozova I, Yu L, Russo JJ, Ju J, Chasin LA. 2011. Quantitative evaluation of all hexamers as exonic splicing elements. *Genome Res.* 21:1360-1374.

Kim S, Shi H, Lee DK, Lis JT. 2003. Specific SR protein-dependent splicing substrates identified through genomic SELEX. *Nucleic Acids Res.* 31:1955-1961.

Lagarde J, Uszczynska-Ratajczak B, Carbonell S, Perez-Lluch S, Abad A, Davis C, Gingeras TR, Frankish A, Harrow J, Guigo R, et al. 2017. High-throughput annotation of full-length long noncoding RNAs with capture long-read sequencing. *Nat Genet* 49:1731-1740.

Longman D, Johnstone IL, Caceres JF. 2000. Functional characterization of SR and SR-related genes in Caenorhabditis elegans. *EMBO J.* 19:1625-1637.

McNally LM, McNally MT. 1998. An RNA splicing enhancer-like sequence is a component of a splicing inhibitor element from Rous sarcoma virus. *Mol. Cell. Biol.* 18:3103-3111.

Niazi F, Valadkhan S. 2012. Computational analysis of functional long noncoding RNAs reveals lack of peptide-coding capacity and parallels with 3' UTRs. *RNA* 18:825-843.

Parmley JL, Chamary JV, Hurst LD. 2006. Evidence for purifying selection against synonymous mutations in mammalian exonic splicing enhancers. *Mol. Biol. Evol.* 23:301-309.

Parmley JL, Urrutia AO, Potrzebowski L, Kaessmann H, Hurst LD. 2007. Splicing and the evolution of proteins in mammals. *PLoS Biol.* 5:e14.

Ramalho RF, Gelfman S, de Souza JE, Ast G, de Souza SJ, Meyer D. 2013. Testing for natural selection in human exonic splicing regulators associated with evolutionary rate shifts. *J. Mol. Evol.* 76:228-239.

Ring HZ, Lis JT. 1994. The SR protein B52/SRp55 is essential for Drosophila development. *Mol. Cell. Biol.* 14:7499-7506.

Sakharkar MK, Perumal BS, Sakharkar KR, Kangueane P. 2005. An analysis on gene architecture in human and mouse genomes. *In silico biology* 5:347-365.

Savisaar R, Hurst LD. 2016. Purifying Selection on Exonic Splice Enhancers in Intronless Genes. *Mol. Biol. Evol.* 33:1396-1418.

Savisaar R, Hurst LD. 2018. Exonic splice regulation imposes strong selection at synonymous sites. *Genome Res.* 28:1442-1454.

Schuler A, Ghanbarian AT, Hurst LD. 2014. Purifying selection on splice-related motifs, not expression level nor RNA folding, explains nearly all constraint on human lincRNAs. *Mol. Biol. Evol.* 31:3164-3183.

Sterne-Weiler T, Howard J, Mort M, Cooper DN, Sanford JR. 2011. Loss of exon identity is a common mechanism of human inherited disease. *Genome Res.* 21:1563-1571.

Tanaka K, Watakabe A, Shimura Y. 1994. Polypurine sequences within a downstream exon function as a splicing enhancer. *Mol. Cell. Biol.* 14:1347-1354.

Wu X, Hurst LD. 2015. Why Selection Might Be Stronger When Populations Are Small: Intron Size and Density Predict within and between-Species Usage of Exonic Splice Associated cis-Motifs. *Mol. Biol. Evol.* 32:1847-1861.

Wu Y, Zhang Y, Zhang J. 2005. Distribution of exonic splicing enhancer elements in human genes. *Genomics* 86:329-336.

Xu R, Teng J, Cooper TA. 1993. The cardiac troponin T alternative exon contains a novel purine-rich positive splicing element. *Mol. Cell. Biol.* 13:3660-3674.

Zhang MQ. 1998. Statistical features of human exons and their flanking regions. *Hum. Mol. Genet.* 7:919-932.
